# This condition impacts every aspect of my life: A survey to understand the experience of living with developmental prosopagnosia

**DOI:** 10.1371/journal.pone.0322469

**Published:** 2025-04-30

**Authors:** Judith Lowes, Lesley M. McGregor, Peter J.B. Hancock, Bradley Duchaine, Anna K. Bobak

**Affiliations:** 1 Psychology Division, Faculty of Natural Sciences, University of Stirling, Stirling, Scotland, United Kingdom; 2 Department of Psychology and Brain Sciences, Dartmouth College, Hanover, New Hampshire, United States of America; Liverpool John Moores University, UNITED KINGDOM OF GREAT BRITAIN AND NORTHERN IRELAND

## Abstract

This mixed methods study examined the real-world experiences of living with developmental prosopagnosia (face blindness), a lifelong neurodevelopmental condition that severely affects the ability to recognise faces despite otherwise normal vision, IQ and memory. Twenty-nine UK based adults with confirmed face recognition difficulties completed an online survey describing and quantifying their experiences of living with poor face recognition. Although the majority (62%) of participants reported being able to recognise their immediate family, e.g., parent, partner, or child, strikingly 35% reported being unable to reliably recognise their immediate family members *out of context*. Even fewer (45%), reported always being able to recognise their three closest friends when encountering them unexpectedly, highlighting that DP commonly affects the recognition of highly familiar faces with whom individuals have close emotional relationships. Furthermore, participants who reported being able or unable to recognise their immediate family showed no significant difference in objective face memory ability. More than two thirds of participants (65.5%) reported being able to recognise fewer than 10 familiar faces (with the most common response being none), far below typical abilities. Thematic framework analysis highlighted how low public, professional, and employer awareness of developmental prosopagnosia presented challenges across multiple domains including seeking diagnosis, social and family relationships and workplaces. Driven largely by concerns about negative evaluation by others, most participants employed a range of highly effortful, though error prone, strategies to disguise and compensate for their face recognition difficulties. Some of the strategies described may help explain why many individuals can perform within typical norms on laboratory face processing tests despite their clear difficulties in everyday life and highlight the need for ecologically valid tests. Participants’ highest priorities for future research were improved awareness of developmental prosopagnosia and interventions to improve their face recognition ability.

## 1 Introduction

Developmental prosopagnosia (DP) is a lifelong condition characterised by a severe difficulty recognising familiar faces, despite otherwise normal vision, IQ, and memory and in the absence of brain damage [1,2]. It can result in severe psychosocial difficulties, including embarrassment, social anxiety, and difficulties in interpersonal relationships [3,4].

Although DP has a hereditary element [5,6] there is currently no genetic test or biomarker for the condition. Instead, diagnosis or classification is usually made based on impaired performance on one or more behavioural face processing tests plus subjective report, following broad guidelines introduced in 2016 [7]. In the UK, and much of the rest of the world, it is almost impossible to obtain a diagnosis of DP from a medical professional [8,9] meaning that individuals seeking an explanation for, or confirmation of, their face recognition difficulties often contact university researchers. However, the choice of tests and cut offs varies widely between research groups (see [10,11] for reviews) in part because the question of which tests, or combinations of tests, are most sensitive for detecting DP remains an open one but also due to the different research questions being addressed.

Recent studies [12–14] reported that around 50% of the individuals who present to research groups with subjective face recognition difficulties show no objective impairments when using the most widely adopted classification approach. These are sometimes referred to as “subjective” [14] or “excluded” [13] DPs. Either these individuals overstate their difficulties or current tests are not sufficiently sensitive. The aim of this study is to catalogue the lived experience of people who have both subjective and objective difficulties. If “subjective” DPs have similar life experience it adds weight to the need for refined objective tests to understand precisely where their difficulties lie.

It is widely reported in the literature that DP is a heterogenous condition both in terms of presentation and severity (see [1] for a review). Although heterogeneity may be due, at least in part, to methodological factors [1], in-depth research with members of the same family revealed different patterns of face processing impairment between family members [15] suggesting that certain sub-processes of face processing may be differentially impaired in cases of DP. Consistent with the latter study and others reporting heterogeneity [16–18], screening interviews undertaken as part of a large study of objective impairment in DP [14] provided anecdotal evidence that individuals with DP can experience the condition quite differently in everyday life and provided motivation to explore this question more systematically. For example, some participants reported that all faces, including those of different ethnicities, were indistinguishable, suggestive of non-identity face perception difficulties. Others however, believed they perceived faces clearly yet forgot the facial identity within minutes, hours or days.

We know much about how such individuals with poor face recognition ability perform on a wide range of lab-based tests (for reviews see, e.g., [1,19]) but so far very little about what relationship, if any, exists between lab-based test scores and real-world experience (for one exception see [20]). A recent scoping review [11] identified that most research published between 1976 and 2022 into DP in adults used lab-based experimental or neuroimaging methods. Of the 224 adult studies identified, only four [4,21–23] employed qualitative approaches, of which two [21,22] were based on the same sample. The review concluded that “it is likely that collection of subjective experiences by participants might open our eyes to as of yet unanswered, and unasked, questions.” [11].

Although DP is a standalone condition, it commonly co-occurs with other neurodevelopmental conditions [24], including object agnosia [19], topographical agnosia [25] and autism [26]. A recent meta-analysis of face recognition abilities in autism [26] found that on face identity recognition tasks the average autistic individual will produce scores lower than around 81% of neurotypical individuals. Additionally, it has been estimated that 36% of the autistic population would meet the clinical cut off for DP [27]. While DP research typically excludes participants with other neurodevelopmental conditions in order to study ‘pure’ DP, evidence that DP commonly occurs with other conditions means that the true proportion of individuals living with severely impaired face recognition is likely to be considerably higher than the commonly proposed estimates of 1–4% of the population with DP alone [28,29] (but see also [10]). Building a better understanding of the experience of living with lifelong face identity recognition difficulties is therefore an important avenue of research in its own right [11] as well as because of the prevalence of severe face recognition difficulties which can create social challenges in those affected.

### 1.1 The present study

The overall purpose of the present study is to generate a better understanding of the range of ways in which the experience of DP manifests in everyday life, and compare the diversity of this experience, from both psychosocial (how does it feel to have difficulties recognising people you know, what is most challenging about living with poor face recognition) and quantitative perspectives. Qualitative insights may identify potential areas of difficulty not tapped by previous testing and could help inform the development of new, sensitive measures for detecting and understanding the underpinnings of DP. The present study also investigates how experiences of face recognition difficulties in everyday life relate to performance on widely used objective tests of face processing. It assesses relationships between the estimates of real-life performance and individuals’ objective face test scores. To our knowledge this is the first to attempt to obtain quantitative estimates of DPs’ face identity recognition ability outside the lab and to investigate and quantify DPs’ experiences of requesting reasonable adjustments or accommodations in the workplace or elsewhere. Such adjustments are increasingly commonly requested, and provided, for other forms of neurodivergence but nothing is known about the experience of requesting these for DP which is currently not classified as a disability and therefore not covered by the UK Equalities Act [30], despite negatively affecting quality of life [4,16]. Finally, this study asks what DPs themselves believe should be the priorities for future research into the condition.

### 1.2 Research questions

Overall, we were interested in how poor face recognition abilities affected individuals in their everyday lives and the diversity of this experience within the DP sample. The following broad research questions were preregistered (https://osf.io/b24ug):

- How many individual faces can people with DP typically recognise and can DPs recognise close friends and family?
- Is there a relationship between real life reported experience (Twenty Item Prosopagnosia Index (PI20) scores [31] and objective Cambridge Face Memory Test (CFMT) [32] scores?
- What aspects of DP do people find most difficult – and conversely are there any positives?
- What is DPs’ experience of seeking a diagnosis?
- What is DP’s experience of disclosing their difficulties to others? Have institutions been willing to introduce adaptations and, if so, have these been helpful?
- What do DPs believe the priorities for future research into DP should be?

## 2 Methods

The protocol and survey questions were preregistered (https://osf.io/b24ug) prior to data collection. Several possible qualitative approaches to data analysis were considered at the pre-registration stage, of these we chose framework analysis which was considered the most suitable once the data were explored.

### 2.1 Context and sampling strategy

Purposive sampling was used to recruit 29 adults (19 women, 10 men), who had previously contacted the research team because of face recognition difficulties. Participants were recruited from two prosopagnosia research groups: 13 from University of Stirling (experimental results from 12 of the 13 are reported in Lowes et al., 2024 [14]) and 16 from Prof. Brad Duchaine’s research group at Dartmouth College, USA. All participants lived in the UK as we wished to examine experiences in a UK context. Objective and subjective face recognition difficulties were confirmed in all participants. Subjective difficulties were assessed using the Prosopagnosia Index (PI20) questionnaire [31], a self-report measure of prosopagnosic traits in real life. Objective face recognition impairment was assessed via four objective standardised face memory measures that were previously used to classify the Stirling cohort; these were the Cambridge Face Memory Test (CFMT) [32], Old New Faces [16] which both measure recognition of newly-learned unfamiliar faces, a score measuring the difference between performance on the CFMT and a matched bicycle memory test (Cambridge Bicycle Memory Test (CBMT) [16]) which assesses the domain specificity of any memory impairments, and a Famous Faces Test (FFT [14]) assessing long-term recognition of facial identities that were familiar to participants. To classify as DP, participants had to score ≥ 61 on the PI20 *and* at least – 1 *SD* below the control age group mean on *two or more* objective tests (with either accuracy or balanced integration score (which adjusts accuracy for response time) as the outcome measure – see Table 2). One additional potential participant showed subjective, but no objective, difficulties and was therefore not invited to complete the survey. Although autism and DP co-occur frequently [26], we included autism (diagnosed or self-diagnosed) in the exclusion criteria since it is likely that autistic traits and experiences could mediate or moderate other experiences of interest, e.g., of social interactions or experiences of seeking healthcare support. Our aim in the first instance was to understand the experience of prosopagnosia in the absence of autism.

**Table 2:** Self-reported face recognition ability score and objective face memory performance.

ID	Age group	PI20	Old New Z	Old New BIS	CBMT Z	CBMT BIS	CFMT Z	CFMT BIS	Faces - Bicycles Z	Faces - Bicycles BIS	FFT Z
DA12	18 - 35	91	-0.19	-2.38	-1.12	-2.20	-1.92	-3.98	-0.79	-1.78	-5.40
DA005	18 - 35	78	0.19	-2.27	0.17	-0.89	0.76	-1.61	0.60	-0.72	0.31
AF017	18 - 35	83	-1.74	-3.17	1.36	2.50	-1.53	-0.17	-2.89	-2.67	-0.04
AF004	18 - 35	68	0.62	-0.67	1.58	0.85	-0.93	-2.20	-2.51	-3.05	-1.87
DA009	36–59	81	-0.28	-3.54	0.82	-2.08	-1.74	-3.75	-2.56	-1.67	-0.01
DA013	36–59	77	-1.94	-3.06	0.39	1.44	-0.03	0.14	-0.43	-1.30	-0.57
DA002	36 - 59	70	-4.72	-6.12	-1.21	-0.62	-2.38	-1.46	-1.18	-0.84	-0.27
AF009	36 - 59	86	0.88	0.03	0.77	-0.85	-1.23	-5.56	-2.00	-4.71	-0.63
DA001	36 - 59	87	-7.50	-8.19	-0.03	0.79	-2.71	-2.30	-2.67	-3.08	-4.42
AF075	36 - 59	72	0.28	-3.07	-0.02	-2.37	-0.80	-3.83	-0.78	-1.46	-1.40
AF001	36 - 59	81	-0.91	-2.45	0.38	1.92	-1.23	-0.35	-1.60	-2.26	-0.24
AF125	36 - 59	92	-0.83	-3.12	1.03	0.05	-1.00	-2.74	-2.03	-2.79	0.43
DA022	36 - 59	95	-0.28	-1.18	0.18	0.61	-2.28	-2.41	-2.46	-3.03	-2.65
AF010	36 - 59	86	-9.84	-12.1	-0.12	0.89	-3.05	-3.48	-2.93	-4.37	-2.63
DA015	36 - 59	82	-4.72	-5.95	0.29	-0.30	-1.64	-1.44	-1.92	-1.14	-2.75
DA020	36 - 59	84	0.28	-0.10	1.35	2.06	-1.21	-1.37	-2.56	-3.44	0.40
DA028	36 - 59	85	-1.39	-3.65	-0.46	-1.74	-1.10	-1.94	-0.64	-0.20	0.25
AF006	36 - 59	95	-2.7	-2.42	-0.41	0.18	-2.09	-1.16	-1.67	-1.34	-1.58
AF003	36 - 59	94	0.88	-0.60	0.87	0.04	-1.55	-2.25	-2.42	-2.29	-1.52
AF060	60 - 74	76	-0.92	-2.94	0.84	0.77	-0.42	-0.07	-1.27	-0.85	-1.92
DA018	60 - 74	86	-3.47	-5.84	-0.63	-3.30	-1.10	-5.17	-0.46	-1.86	-5.27
DA033	60 - 74	77	-2.80	-3.23	0.01	-0.52	-0.26	-0.23	-0.28	0.29	-1.68
DA014	60 - 74	87	-8.80	-9.60	-0.73	-1.05	-2.95	-2.89	-2.22	-1.84	-4.67
DA017	60 - 74	92	0.53	0.03	-0.17	0.08	-1.47	-1.74	-1.30	-1.82	1.40
DA004	60 - 74	75	-3.47	-3.59	1.22	1.01	-0.45	-0.55	-1.67	-1.56	-3.22
AF019	60 - 74	99	-3.88	-6.92	0.84	0.02	-1.64	-1.72	-2.48	-1.74	-5.22
DA006	60 - 74	71	-0.80	-1.62	0.85	0.58	-0.45	-0.37	-1.30	-0.95	0.07
AF099	60 - 74	74	-3.14	-7.53	0.09	0.11	0.33	0.22	0.23	0.11	NA
AF008	60 - 74	77	-0.17	-5.72	0.84	-2.19	-0.52	-1.23	-1.36	0.96	-1.37

*Note*: PI20 = Prosopagnosia Index 20 score; Old New = Old New Faces Test; CBMT = Cambridge Bicycle Memory Test; CFMT = Cambridge Face Memory Test; FFT = Famous Faces Test.

Z scores are accuracy scores centred on the relevant age-group control means thus comparing each participant to typical controls of a similar age. BIS = Balanced integration scores which are calculated as Z_accuracy_ *minus* Z_RT_ using RT on correct trials only.

Thus, we investigate the experience of a cohort of individuals (DPs) who meet the widely accepted definition of DP, showing both subjective and objective face recognition difficulties. Our study does not aim to provide a representative sample of DPs. Sample size was guided primarily by resource limitations: The maximum number of potential participants is constrained by the number of qualifying individuals with prosopagnosia already known to the researchers and time constraints. We initially anticipated 30 participants; 31 eligible participants started the survey but two did not complete it, leaving a total of 29 complete responses.

### 2.2 Ethical issues pertaining to human subjects

Approval for the study was granted by the University of Stirling General University Ethics Panel (reference 14876). All participants provided informed written consent, including for the use of de-identified quotes, and research was conducted in accordance with the principles of the Declaration of Helsinki. Data were collected using unique identifiers.

### 2.3 Data collection methods and instruments

Data were collected using a mixed methods approach. Participants completed an online survey comprising a combination of open response, multiple choice, and closed response questions. The qualitative questions were designed to encourage detailed responses, and many qualitative, but also quantitative questions, were informed by information provided by potential DPs when they first contacted us. Data collection ran from 23 August 2023–21 February 2024 using the Online Surveys platform (www.onlinesurveys.ac.uk). Participants received a £5 gift voucher upon completion. A draft survey was piloted with two prosopagnosic participants using a “think aloud” process. Based on the resulting feedback and suggestions, several questions were reworded to improve clarity. All participants reported here completed the same final survey and questions are provided in S1 Table. The survey typically took between 20 and 59 minutes to complete.

### 2.4 Participant characteristics

Participant demographics are shown in Table 1 below. Participants (*N* = 29; 19 female, 10 male) were aged between 26 and 73 years (M_age_ 53.5 ± 13.8). All participants reported normal, or corrected-to-normal, vision, no neurodevelopmental or psychiatric conditions (other than mild dyslexia), and no history of brain injury. However, participants were not scanned to confirm their reports. Three respondents provided somewhat ambiguous responses to specific questions about the onset of their face identity recognition (FIR) difficulties but were included in the analysis since they had all previously confirmed that their FIR issues were lifelong and none of their qualitative responses suggested sudden onset.

**Table 1:** Participant characteristics.

ID	Age Group	Gender	Employment status	Family history of face recognition difficulties?	PI20 score
DA012	16-35	Female	Working	Yes	91
DA005	16-35	Female	Not working due to illness, disability or family reasons	Yes	78
AF017	16-35	Female	Working	Yes	83
AF004	16-35	Male	Working	Yes	68
DA009	36-59	Female	Working	Don’t know	81
DA013	36-59	Male	Working	Don’t know	77
DA002	36-59	Female	Working	No	70
AF009	36-59	Male	Working	Yes	86
DA001	36-59	Female	Working	Yes	87
AF075	36-59	Female	Unemployed but available to work	No	72
AF125	36-59	Female	Not working due to illness, disability or family reasons	No	92
AF001	36-59	Female	Working	No	81
DA022	36-59	Female	Working	Yes	95
DA015	36-59	Male	Unemployed but available to work	No	82
DA028	36-59	Male	Working	No	85
AF010	36-59	Female	Working	Don’t know	86
AF006	36-59	Female	Working	Yes	95
DA020	36-59	Female	Working	No	84
AF003	36-59	Female	Working	Yes	94
AF060	60-74	Female	Retired	No	76
DA018	60-74	Female	Retired	No	86
DA033	60-74	Female	Working	Yes	77
DA014	60-74	Male	Retired	No	87
DA017	60-74	Female	Retired	No	67
DA004	60-74	Male	Working	Don’t know	75
DA006	60-74	Male	Retired	No	71
AF019	60-74	Male	Retired	Don’t know	99
AF099	60-74	Male	Retired	Yes	74
AF008	60-74	Female	Retired	Yes	77

*Note*: The PI20 [31] is a validated questionnaire measuring self-reported prosopagnosic traits; higher scores indicate more difficulties and the cut off used was 61. Participant IDs beginning AF were recruited via Stirling and DA via Dartmouth. Participants are listed by ascending age and grouped by age group.

### 2.5 Data processing

Where a participant provided a range for their numerical estimates, the median value was input (for a range between 10 and 20, the median would be 15). Face memory test scores were standardised and centred on control age group means collected as part of a previous prosopagnosia classification study (see [14] for a detailed description of the control sample and approach).

### 2.6 Data analysis qualitative approach

Qualitative survey data were analysed using framework analysis (FA), a five-stage approach to thematic analysis described in Spencer & Ritchie [33] that is suitable for both contextual and diagnostic research questions. FA allows codes to be generated both deductively (e.g., from theoretical models of face processing) and inductively (from questions). FA was chosen for several reasons. First, it enables data to be analysed systematically yet flexibly and crucially it also allows *between*-case and *within*-case analysis thus allowing individual patterns of responses to be assessed and compared [33]. Second, FA permits us to analyse the diversity of DPs’ experience without prioritising, giving - or appearing to give - additional weight to more common responses (cf. content analysis). Our aim is not to describe a “typical” experience of DP but rather to capture the richness and diversity of lived experience. In line with Pyett [34], we consider each person’s experience to be valid and potentially informative, regardless of whether it is typical of the wider cohort, or not. In neuropsychology, there is a long history of single case studies that have resulted in important theoretical insights and therefore an individual’s experience, or pattern of responses, does not need to be widely found to have potentially important theoretical and/or explanatory value. Third, we wanted to be able to consider lived experience in the context of each individual’s objective performance on face memory tests and FA’s use of charts to analyse between-participant responses across multiple questions and measures is well suited to this task.

We used NVivo 14 [35] to analyse qualitative data. Open responses were initially coded inductively, and we next developed some additional deductive codes. The survey questions themselves were used deductively to develop the charting; charting was then expanded to incorporate the key inductive themes that were developed from the coding of individual survey responses. Some quotes in the text have been edited to correct spelling mistakes and to change details which might identify participants. Quantitative data were explored via descriptive statistics, independent samples t tests (or non-parametric alternatives) and correlational analysis using Jamovi [36].

### 2.7 Techniques to enhance trustworthiness

FA is considered a type of ‘codebook’ thematic analysis for which inter-rater reliability and coder consensus are not considered appropriate quality indicators [37]. JL conducted the primary coding, charting, and thematic analysis and kept a reflexive log to track the development of codes and themes and to document key decisions and how potential bias was addressed. At each stage of the analysis, JL discussed coding and analysis decisions with LM, an experienced qualitative health psychology researcher with no experience of prosopagnosia research who thus brought a different perspective and had no prior expectations informed by current face processing theory. LM independently coded four responses; coding differences and similarities were then discussed. From this pilot work, the final coding framework was agreed and applied. Thematic decisions were reached through consensus within the author team that includes three additional face researchers.

### 2.8 Researcher characteristics and reflexivity

JL, BD, and AB have previously researched prosopagnosia and PH is an experienced face researcher. LM is an experienced health psychologist with research expertise in qualitative methods and the lived experience of health conditions. None of the research team has prosopagnosia. The sampling frame for this study is individuals who contacted one of two prosopagnosia research groups because of severe face recognition difficulties who had given consent to be contacted about future research studies. JL previously conducted screening interviews by telephone or video call with some participants (*n* = 12) to confirm their eligibility for a prior experimental face recognition study and exchanged emails and administered online testing with the remainder (*n* = 17). No diagnosis was provided to participants although a small number of participants requested their test scores which were supplied after their survey responses were received.

## 3 Quantitative results

### 3.1 How many individual faces can people with DP typically recognise?

Participants were asked to make a private list of the individuals they believed they could reliably recognise *out of context* and to provide the total number of these identities. Estimates varied widely [range 0–2500], the mode was zero, and the median seven. Overall, 69% of participants (20/29) reported being able to recognise fewer than 10 familiar faces which is far below the estimate of an average of 5000 faces that a typical adult can recognise [38]. Although these studies use different methods to arrive at these estimates, the difference is nonetheless striking. Note that the participant who reported being able to recognise 2500 faces told us he reached this estimate by totalling groups of people he believed he could recognise (e.g., school friends, colleagues, family) rather than listing and totalling individual names.

### 3.2 Can DPs recognise close friends and family?

**Fig 1:**
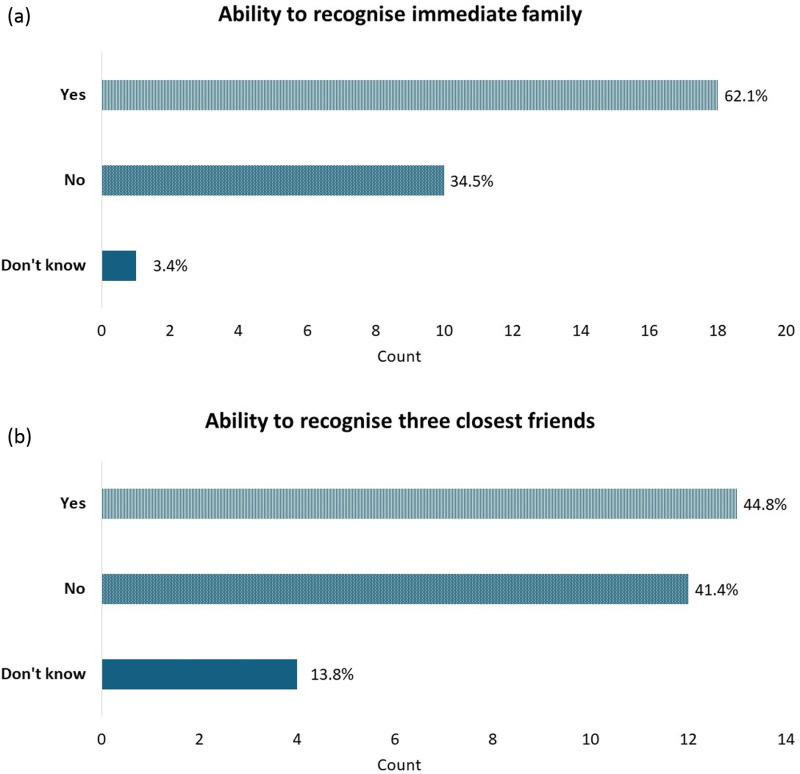
Ability to recognise immediate family and close friends if meeting them unexpectedly out of context.

As shown in Fig 1, although the majority (62%) of participants reported being able to recognise their immediate family, e.g., parent, partner, or child, strikingly 35% were unable to reliably recognise their immediate family members *out of context*. Even fewer, 45%, reported always being able to recognise their three closest friends when encountering them unexpectedly. Chi-square or Fisher’s exact tests showed that these responses did not differ between genders, age groups, or recruiting university (all *p*s >.09). Qualitative findings and illustrative examples of difficulties recognising family are discussed below in subtheme 3.3.

**Fig 2:**
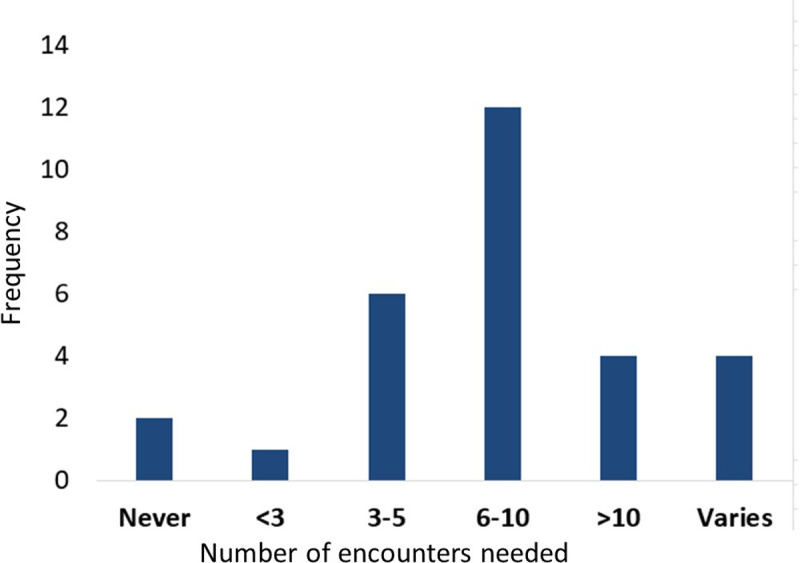
Number of encounters DPs needed to be able to recognise a person.

When asked how many times participants typically needed to meet someone before they might recognise them, responses again varied widely (Fig 2). At one extreme, one participant reported being able to recognise others after only one or two encounters, although they cautioned that recognition was only possible if they had been able to make an effort to memorise something distinctive about the individual and associate this with their name. This type of deliberative approach is very different to that used by typical perceivers. At the other extreme, two participants reported *never* being able to recognise a face, regardless of the number of encounters. Overall, the most common response was between six and ten times. Many participants noted that they found some individuals much easier to recognise again than others. Sometimes this depended on whether the person had distinguishing features, for others it was due to the “intensity” or “recency” of the encounter, or for reasons participants could not articulate.

### 3.3 Relationship between real life experience and test scores

Next, we asked whether there was a relationship between real life reported experiences, e.g., the ability to recognise immediate family or the number of facial identities participants said they could recognise, and standardised objective face memory measures or PI20 scores shown in Table 2. The control scores on which z scores were calculated are shown in Table 3.

**Table 3:** Control performance used to calculate z scores.

Proportion correct	CFMT			CBMT			Old New			FFT		
	*N*	*M*	*SD*	*N*	*M*	*SD*	*N*	*M*	*SD*	*N*	*M*	*SD*
14 - 35 years	21	.81	0.14	21	.81	0.12	21	.95	.09	23	.93	0.09
36 - 59 years	20	.81	0.13	22	.81	0.14	19	.95	.06	22	.89	0.12
60 - 74 years	17	.72	0.15	18	.78	0.15	17	.94	.05	18	.86	0.10

*Note*: CFMT = Cambridge Face Memory Test; CBMT = Cambridge Bicycle Memory Test; Old New = Old New Faces Test; FFT = Famous Faces Test. Control scores are taken from our previous DP classification study [14].

Independent *t* tests (see S3 Table) showed that objective face memory scores and PI20 scores were not significantly different between the participants who reported being *able* or *unable* to recognise immediate family, all *p*s >.172 and all Bayes Factors < 0.80).

Researchers sometimes classify DP as “mild” (typically using z scores ≤ -1 on two separate objective measures) or “major” (z scores ≤ -2 on two separate measures). Using this approach to classification, it was noteworthy that the proportion of participants who reported being unable to recognise close friends or immediate family (or both) was fairly similar for both groups; “mild DPs” 42.9% (3/7) “Major DPs” 54.6% (12/22). In other words, laboratory measures of face memory showed little relationship with the severity of everyday experiences within this DP sample. Consistent with this, Spearman correlational analysis showed no significant associations between the number of familiar facial identities that participants estimated they could recognize in, or out of, context and any of the test or questionnaire scores (see S2 Table).

Although CFMT control accuracy was lower in the older age group, thus potentially making it more difficult for older DPs to produce significantly different z scores vs controls, control mean accuracy among 60–74 year olds was.72 [.15] thus well above chance (.33) and also above what might be considered floor performance (.50, i.e., a score of 36/72 which is full accuracy on the 18 introductory CFMT items and chance performance on the remaining 54 items). Range restriction cannot therefore fully account for the 70% of DPs who scored within 2 SD of control mean CFMT accuracy on this task. It should be noted that some of the variability in impaired scores, in DPs and arguably also in controls, will reflect factors other than face recognition ability, e.g., motivation.

Due to participant error AF099 did not complete all the objective classification tasks but is classified as DP as due to a very low z score on the old new face recognition task.

### 3.4 What types of face recognition errors do DPs make?

**Fig 3:**
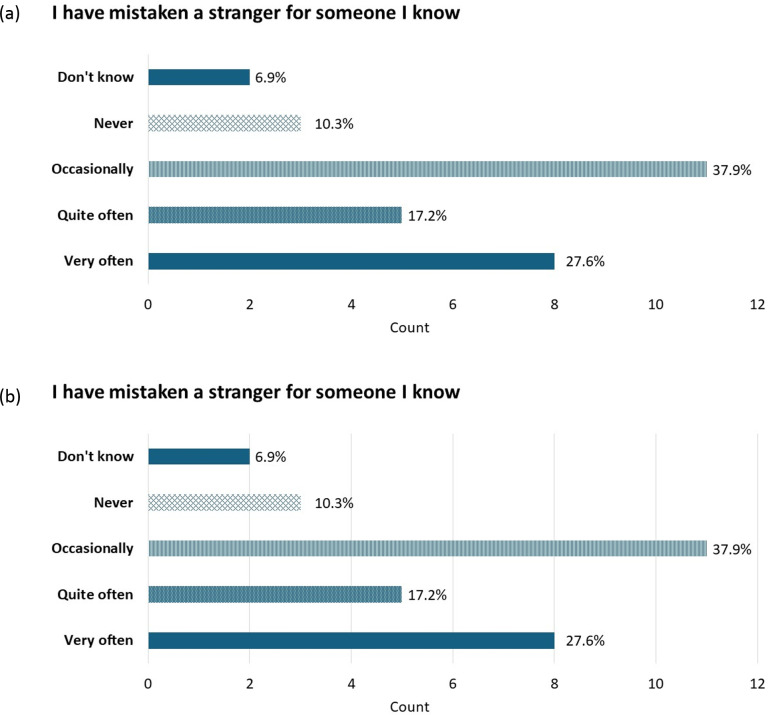
Frequency of error types. *Note*: Bars show the count of respondents (maximum 29) who selected each answer option, with the percentages shown at the end of each bar.

Unsurprisingly, within this sample, the types of errors that participants reported making varied widely (Fig 3). Forgetting previously recognised familiar faces was a common, though not universal, experience. False alarms (mistaking a stranger for a familiar person) were relatively less common, though still widespread, with only a small number (*n* = 3, or 10%) of participants reporting never making such errors Fig 3.

## 4 Qualitative analysis of the written narratives of living with DP

Five themes were identified inductively and deductively from the open-ended responses, and themes and sub-themes are shown in Fig 5 below. A full list of the codes, mapped to themes is provided in S5 Table.

**Fig 4:**
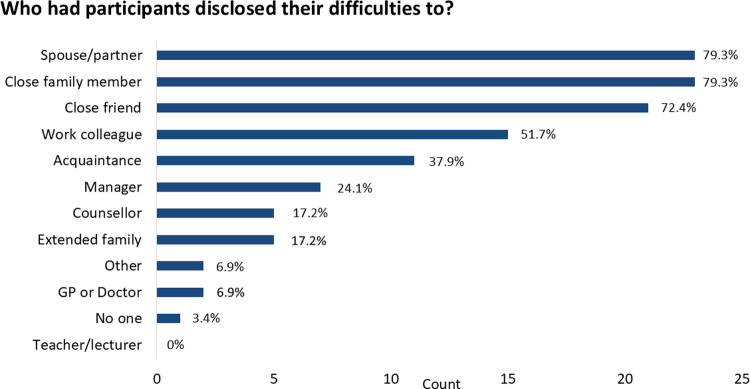
Who had participants disclosed their face recognition difficulties to? *Note*: Bars show the count of respondents (maximum 29) who selected each answer option. Participants could select more than one option. Participants did not report relationship status so it is possible some participants have not been in a relationship since they became aware of their difficulties with face recognition.

### 4.1 Theme 1: Seeking explanation, validation, and diagnosis

This theme describes the process by which participants came to realise their face recognition difficulties were atypical, and the existence of DP as a condition. It captures the sources of information participants sought out to understand or explain their difficulties, and their experiences of seeking a diagnosis (or classification) whether formal, informal, or self-diagnosis and how this made them feel.

#### 4.1.1 Sub theme 1.1 “*I thought it was just me*”.

Although all participants were aware that they struggled to recognise people they knew and had poor face recognition, they had usually been unaware of the existence of DP as a condition for many years. Instead, they often believed their difficulties were “*my own fault*,” unique to them, or a result of “*some inadequacy.*” Several participants reported that their awareness that DP is a scientifically and, in some cases, medically recognised condition [39] came relatively late in life, for example, one participant was in their sixties and others were in their forties.

*“I…found a few websites with interesting information, which helped me understand my difficulties were real, and not just something only I experienced, or imagined I experienced.”* (AF009)

*“I have only been aware of…face blindness properly for only a few years, I didn’t know it was a thing.”* (DA028)

Apart from one participant who was ‘diagnosed’ by fellow medical students during a lecture covering prosopagnosia, it was much more common for participants to discover the existence of DP or face blindness for themselves. Some described a “*light bulb*” moment when they heard or read about prosopagnosia and recognised their experiences in the description. Often this was though media coverage, a podcast, or - less commonly – as a result of studying psychology or reading a novel featuring a character with face blindness [40]. Most participants described using the internet both to seek an explanation for their face recognition difficulties *(“Dr Google*”) and to seek further information about DP once they were aware of what to search for.

#### 4.1.2 Sub theme 1.2 The struggle to find reliable information.

Almost all (*n* = 27) participants had tried to find information and advice on DP. We enquired about sources and particular sites that participants had used to find out more about DP once they knew about the condition. The internet as the main source consulted but relatively few participants could recollect specific sites. Several participants mentioned Facebook self-help/support groups. The most mentioned information sources that participants could remember accessing belonged to university research groups, in particular faceblind.org, a site run by Prof. Brad Duchaine at Dartmouth College, USA and the support organisation Faceblind UK. Three participants had looked for information on the NHS England website, however two reported they found the information provided there to be of limited value and “*very basic*.” (DA001).

#### 4.1.3 Sub theme 1.3 Perceived value in diagnosis.

Obtaining a formal diagnosis did not initially appear to be important to all participants, only 34% (10/29) had sought testing or diagnosis. One possible explanation is that several participants noted that that [in the UK] there is currently no treatment or support for DP and believed that UK health services have limited awareness and expertise around the condition. As a result, some participants were uncertain about the value of a diagnosis and/or where to seek support:

*“I have never discussed it with a medical professional (and my understanding is that it would be hard to access one who knew as much as I do about the condition).”* (AF003)

*“I didn’t feel like there was anywhere I could go. The NHS website just describes it but doesn’t say what to do next.”* (DA013)

A minority of participants (*n* = 7) reported that family or friends had suggested they get tested for prosopagnosia, but it was not clear from responses whether these suggestions were offered after participants shared their own suspicions or concerns about their face recognition abilities, or whether this was unprompted advice.

Despite our sampling method, only one participant had received a formal diagnosis (from a researcher) prior to our study. Three more had been told by researchers they met the classification for DP, for them this constituted a diagnosis. A minority were keen to be formally diagnosed themselves; they felt diagnosis could be helpful for achieving some recognition of the challenges they experience as well as providing an explanation to others that, crucially, would be accepted. Even when not formally diagnosed, self-diagnosis through completion of an online test or simply recognising oneself in a description of DP allowed participants to give a name to their difficulties. Learning of the existence of DP as a condition appeared to be very important to many participants for whom the realisation that their difficulties were due to a recognised condition brought relief, validation, and a welcome explanation for why they found recognising people so difficult. Only one participant had approached a GP to request testing for possible developmental prosopagnosia; they reported the GP *“shrugged [their] shoulders”* and said it was *“not possible to test for it and not much you can do about it.”*

We enquired whether participants had disclosed their difficulties to others (separate from seeking a diagnosis). As shown in Fig 4, only a small minority (6.9%) had discussed their face recognition difficulties with a doctor, and only 24% had told their manager. Even more strikingly, around 20% (6/29) had not disclosed their difficulties even to immediate family (a similar proportion had not told an intimate partner although it is possible some participants have not been in a relationship since they became aware of their difficulties with face recognition). No participant reported discussing their difficulties with a teacher, even though at least two were keenly aware of their difficulties while still at school.

Even within *families*, diagnosed or suspected DP was not always openly discussed. Seven participants knew of close family members who also had very poor face recognition, and one respondent believed her young daughter “*might also have issues with faces*.” Two participants mentioned that they only become aware of their own parent’s face recognition difficulty after disclosing their own difficulties. Given that many participants described researching prosopagnosia extensively online, it might have been expected that they were also aware that DP often has a hereditary link, so it was initially surprising to us that many participants reported not knowing whether other family members were affected. This lack of knowledge of family members’ difficulties, despite information online about the hereditary nature of DP, is consistent with the finding that relatively few (5/29 or 17.2%) reported having told any of their extended family that they had difficulty recognising familiar faces (Fig 4) indicating that the topic is not something most participants appeared to feel comfortable or able to bring up, even among extended family, perhaps due to perceived stigma or self-stigma.

### 4.2 Theme 2: “You don’t know how difficult it is navigating life as a prosopagnosic”

Theme two captures depictions of what it’s like to live with poor face recognition, including whether this changed over time – for example whether certain life stages were more difficult and if the condition gets more, or less, challenging with age. It describes the types of settings and circumstances that participants said they found the hardest and captures the techniques participants use in their everyday lives to try to recognise people as well as the extent to which they find these strategies helpful. We did not want to assume that the effects of DP are always negative, so we enquired about any perceived benefits or advantages of the condition. The theme captures participants’ beliefs about DP and how these affected their self-identity.

#### 4.2.1 Sub theme 2.1 The most challenging aspects of DP.

We asked participants what they found most challenging about having poor face recognition. Reponses tended to highlight either situational factors, discussed here, or the emotional impacts which are reported below. Meeting people out of context was commonly reported as challenging, as were situations where large numbers of people were present, for example conferences, parties, and large social events:

*“My daughter started school last year and having to meet 30 new kids and 60 new parents was pretty much my worst nightmare!”* (DA001)

*“Large rooms are hard, it’s a bit Where’s Wally. Everyone merges into non people.”* (DA012)

For most participants, DP did not solely affect their ability to recognise acquaintances or individuals they had only met briefly. Rather, it also caused considerable difficulties recognising people with whom they had close relationships and/or saw very frequently including office colleagues, close friends, neighbours and family:

“*I could easily walk past pretty much anyone I knew except possibly my husband and children. It is isolating and exhausting living in a world of strangers.”* (AF125)

“*Sometimes when I am off work for a week and come back it’s really hard to* figure *out who is who*.” (AF017)

*“If there are lots of people around it is particularly challenging to even find a familiar face. For instance, in a shopping centre or airport with my husband I will ask him to wait in a specific place if I visit the loo so that I can find him when I come out…”* (AF009)

Work was frequently mentioned as a particularly challenging setting. Reasons given included the need to interact with relatively large numbers of people, for example in universities, schools, hospitals, or business consultancies where teams and clients changed regularly. In some work settings, uniforms were worn thus removing cues that participants often relied on such as clothing and accessories. For others, the high cognitive demands of work prevented the use of the deliberative, associative face learning and recognition strategies they usually relied on.

*“If my brain is busy and I’m focusing on multiple other things at the time of meeting someone I may struggle to recognise them later that same day…If I’ve been introduced to multiple people at one time I might struggle to identify them later on. This is particularly severe if…trying to retain other information relating to the job I’m doing or working on something particularly complicated*.” (AF004)

Although most participants provided detailed accounts of settings, or circumstances, they found particularly difficult, a small minority (*n* = 2) initially reported (in response to a direct question) that their face recognition difficulties cause no major challenges in their life. Despite this, both later reported embarrassment during social encounters, with one going as far as to say that when they do not recognise someone they know “*Through the eyes of person who recognises me it most likely devalues what they considered to be a close relationship and can actually change the way in which they think of you but in a negative way*” (DA014).

#### 4.2.2 Sub theme 2.2 The use of effortful strategies for recognition.

We enquired about the strategies people used to recognise others. Spontaneous responses are categorised in Table 4 below alongside examples of how and when strategies can fail. After participants provided spontaneous responses, they were then provided with a list of strategies that DPs had previously described using and asked to indicate if they had ever tried them, and, if so, to rate how useful they found each strategy (Fig 6 below).

**Table 4:** Compensatory strategies (spontaneous report).

	Examples of strategy use	Examples of strategy failures
**Situational context** (e.g., where a person sits at work or in an orchestra	“Think about who I expect to be present and work by process of elimination.” “Context and expectation is everything…if I know someone would be there, then I stand a much better chance of recognising them.”	“It’s hardest when the situation is entirely out of context - school mum in supermarket.” “…at a wedding or similar multi-generational event - where some people are my friends, some are friends of parents that I’ve known from growing up…there’s no context around anyone…”
**Context derived from conversational cues**	“When I met someone that it is clear I should recognise but don’t, I tend to play along with generalised comments and fish for clues as to context and identity.”	
**Voice**	“Accents help” “Voice - I’m good at recognising voices and am usually fine once someone speaks.”	“Voice is no good as a recognition cue, because I don’t recognise voices any better than faces” “If the person is not speaking, I lose another clue that I use to recognise people”. “Starting a new job when I know I will meet many new people within a short space of time. I won’t have had time to learn their voices or other things that would help me to tell them apart.”
**Body** (gait, posture, shape, build)	“Look at their overall body profile, height & build” “Some people have a distinctive walk”	“Meetings where people are seated are harder than standing-up occasions because it’s harder to use, e.g., gait clues
**Hair** (including hair colour, style, length, facial hair, or baldness)	“I attempt to remember things like hair colour and style…”	“When someone changes something like their hair etc it can be a challenge.”
**Mannerisms**	“Oddly enough, in my year at school, we had a pair of identical twins. My friends told me they couldn’t tell the twins apart, but I found it rather easy. I can’t quite put into words why…I suspect it was down to their mannerisms. I guess my friends focused on other details than I did to ID the twins.”	
**Memorise facial features** or style of make up	“Memorising distinctive facial features, e.g., thick eyebrows, shape of chin etc.” “Distinctive unsightly facial features, e.g., scars”	“Due to the nature of my work I’m often in dark venues or on dark stages and therefore unable to pick out particular features.”
**Memorise extra-facial features** (glasses, accessories, tattoos, piercings, teeth, ears)	“Distinctive features - tattoos or piercings are a good thing to look out for as they typically do not change” “By trying to remember something specific such as a piece of jewellery…”	“It’s more challenging when…you meet them with/without glasses…”
**Smell**	“Sometimes smell helps, which sounds weird but if someone has a strong aftershave or perfume it can help me to identify that person.”	
**Things people typically have with them**	“They might also have a baby or toddler with them in a pram/buggy that I recognise” “Their dogs, cars, musical instruments”	“The other day I got chatting to a man whom I presumed I had met before because I knew his dog. It turned out it was his wife who had been walking the dog when I had met it several times before. I was surprised that I had forgotten the sex of the person as well as the face.”
**Wait for others to make the first move**	“When agreeing to meet a friend (e.g., for coffee), I often wait for them to greet me.” “If I see someone I THINK I recognise, I don’t acknowledge them or make eye contact until they make the first move.”	
**Name prompts**, e.g., name badges, online meeting technology	“When I was working and people wore a security pass I tried to glance at that to see their name.” “During the pandemic it was a lot easier, since on Zoom the names were always displayed, and I think I relied too much on these and focused even less on how people look.”	“Name badges are really helpful but often small and difficult to read or really obvious that you’re looking at them when you should know somebody.” “Online meetings where… Zoom etc…would tell you who everyone was were great! I am really sad that so many meetings have gone back to being in person.”
**Clothing** or clothing style, including shoes	“Clothes they characteristically wear…” “Remember clearly looking at people’s shoes at school to know who they were.”	“I have trouble following plots in films because as soon as the scene changes, and the actor’s clothes have changed, I struggle to recognise whether it is the same person or a new character.” “I work in an area where there are lots of people wearing the same attire and this can be especially difficult. Also as it is in healthcare and my patients change into gowns I can’t recall who they are.” “I recognised one particular mum by a specific coat (a red puffer jacket) she wore every day in the winter. But when it came to the spring term, she no longer wore the coat so I couldn’t recognise her. Then even worse... I found out that there were TWO mums with the same coat, and I’d been regularly calling one of them by the other’s name, not realising they were two different people, aargh!”
**Use of written notes**	“I used to work in a large supermarket, so I made an excel spreadsheet of names/ hair colour/hair length/distinguishing features/their typical greeting/hobbies to tell people apart. I kept it on my phone so it was to hand when I needed it.” “When teaching, I ask my class to introduce themselves the first time and draw a plan of who’s sitting where and draw sketches of their bag while they’re doing an activity later. They tend to sit in the same spaces, or next to the same people, in subsequent lessons.”	
**Prompts from others**	“I have to rely on my children to prompt me and, again, that makes me feel stupid.” “In a work context (e.g., meeting clients) I often struggle. When working with other members of my team I brief them in advance to introduce themselves if they haven’t met the client before, so that I can have firm confirmation that it’s the right person!”	“I usually just ask my husband who they are. If he’s not there, then I just act politely and keep the conversation vague and then feel bad afterwards for not remembering who it was.” “Asking my wife to prompt me when she knows I do know someone is best, if not always practical.”
**Other strategies**	“I have severe difficulty identifying people out of context, when I generally can’t say whether I know them or not; but I think I can often tell by the way they look at me. I cultivate a unique personal appearance, which makes people more likely to look and give me a clue as to whether they recognise me or not!” “Quite often, person X will remind me of person Y and I’ll remember that fact, which will happen reliably even if I don’t know why…” “Going to the football is another time when I notice I have more difficulties than other people, I couldn’t recognise the players I watched every week even when I was going to most games. I always had to rely on the shirt numbers and their position on the pitch to try and make an educated guess…except for players [who were] particularly distinctive.”	“(Of course that’s only useful if I am not going to see X and Y in the same context! Unfortunately, our new Head of School reminds me strongly of an existing colleague...)”

The complexity of strategies and approaches described highlights the sheer level of “*conscious effort*” that many participants were required to make to try to recognise familiar people, a task that those with typical face recognition ability can achieve rapidly and effortlessly. Participants were usually aware that some strategies were more reliable for them than others, and that particular approaches often failed (particularly reliance on clothing, situational context, and hair). As a result, participants often adopted a staged approach whereby they developed a range of potential strategies and tried them out either in combination, or in turn:

*“It’s more helpful to learn the things less likely to change often, e.g., voice> tattoos> body shape> hair colour> hairstyle>...and then clothing etc. is less helpful in a long-term sense.”* (DA001)

This participant emphasised that sometimes it was the *combination* of cues that most aided recognition:

*“…it’s easier to recognise a couple (ah yes, she’s the petite athletic-looking Asian lady, who has a very tall white husband with a beard, etc.) because the combination of features is more unique.”* (DA001)

Although these atypical strategies could often assist with person identification, it was not always possible to employ them (see second column, Table 4). Furthermore, many participants reported that they found their strategies were much less helpful when meeting multiple people at the same time, perhaps explaining why so many participants said they found group settings particularly challenging. The strategies were also reported by many to be more helpful in the short, rather than the medium or longer term:

“*I can do a sort of “emergency recognise”, where I memorise all the shapes of the facial features in the same way you would with a memory game (e.g., memorising objects in a room etc.) It only lasts for an hour or two, and if I encountered them again in the future, I’d have no better chance of recognising them than anyone else.”* (AF006)

Participants’ regular, embarrassing failures of face recognition were not due to lack of trying. All participants reported having tried at least four different strategies [median = 8, range 4–13] with varying degrees of success (Fig 6). For example, the use of written notes was mentioned by 28% of participants and found helpful by 100% of those who used it; this took the form of making detailed notes about appearance, mannerisms, characteristics, typical greetings, and hobbies, as well as or sketching bags or typical seating plans. Additionally, several participants described actively “revising” their notes in advance of expected encounters.

Most participants described using conscious, deliberative strategies to assist with identification. However, one participant reported that their strategy use was relatively unconscious, at least initially *“I think I’ve always used these strategies even before I knew about prosopagnosia”* (AF010) and another wrote that they had not realised that they were using any strategies until they read the list of possible strategies that others had reported using.

Less common, but interesting, recognition techniques included memorising, or keeping a record of, the typical greetings or idiosyncratic phrases that people use and a person’s smell (e.g., a distinctive fragrance worn). The increasing use of online meetings in both social and work contexts was mentioned by several participants as helpful, both because names appear beneath each face and because virtual meetings provide an opportunity to take screenshots of faces with names attached that can be used for future reference.

#### 4.2.3 Sub theme 2.3 Differing beliefs about DP and its impact.

Several participants highlighted how, with hindsight, their face recognition difficulties have made certain life stages relatively easier or more difficult for them. A few participants felt that their condition affected them more as they aged, either because as “*a youngster I was more frequently going out with friends and now realize I used to rely on them to “remind” me who someone was*” (AF099) or because the number of people one is “*expected to”* recognise increases with age (AF003). The natural decline of memory in older adulthood was also highlighted as a factor by two participants. For one, this was because poorer general memory made it more difficult to implement their previously successful strategy of memorising features. By contrast, another felt they were more likely to be misjudged as “*suffering from dementia (now that I’m a bit older*)” (DA018).

Interestingly, one participant felt her face recognition abilities might have a hormonal link:

*“This might sound slightly crazy, but …I find that I’m slightly better at recognising people during certain times of the month. Also whilst I was pregnant I went through a couple of months where I thought I recognised absolutely EVERYONE! That strong “I’m sure I know you from somewhere” feeling. (It felt like a super power, even though I’m sure I was 99% wrong, haha).”* (DA001)

Over time, some participants reflected that they gained some acceptance either of the condition itself as “*a limitation that I can do very little about*” (DA020); or of the discomfort it causes during social interactions:

*“…learning to accept the awkwardness of that second meeting where I am unaware of who I am talking to and just plodding on with the conversation anyway has also helped, as I will often remember who the person is eventually…”* (AF099)

Although neither the participant information, nor the survey questions, framed DP as a disability or a form of neurodiversity or neurodivergence, it was notable that around 20% (6/29) of participants used these terms themselves when describing their experience, indicating that they had considered whether DP should be categorised in these terms. These participants expressed mixed views on whether they themselves considered DP a disability, but frequently commented that, despite the severe challenges DP presents, UK society does not currently recognise DP as a disability.

“*I tend not to think of it as a disability, more an eccentricity.”* (DA033)

*“I don’t have a recognised disability but, also, I can’t do what other people can do so I’m at a huge disadvantage…”* (AF125)

*“I like to think of it as a superpower rather than a disability. But if we class it as a disability, it’s a hidden disability. People don’t realise you’re faceblind unless you tell them, and it’s usually not on their radar…”* (DA012)

Others described DP, both directly and indirectly, as a form of neurodiversity. One participant expressed the wish that DP could be “ *recognised by HR professionals as on a par with neurodiversity*” (DA006) with another stating that “*It needs to be seen as part of a spectrum of human abilities and not a disease.*” (DA022)

#### 4.2.4 Sub theme 2.4 Silver linings.

We enquired about what positives, if any, DP brought. One participant reported meeting his now wife as a result of mistaking her for someone else “*something I am always teased about*” (DA014)

Fascinatingly, three separate participants (DA012, DA015, AF019) noted that they could easily tell identical twins apart when their friends could not. Other participants worked with clients in sensitive confidential settings and found some occasional benefits to having DP.

*“It is actually weirdly handy for my work role. I work face-to-face with vulnerable clients in the community and I know I will be unlikely to have that awkward moment of bumping into service users out and about.”* (AF017)

An academic reported a similar benefit since, for example, they would never remember which student they had needed to tell off. A small number of participants felt that they had been able to turn some difficulties that DP presented into positives. For example, attentive listening (due to the need to seek conversational cues to identity), an ability to “*pivot conversations*” easily (when suddenly realising the person they were speaking to was not the person they had initially thought it was), and public speaking in front of strangers (all or most people look like a stranger to them). One participant wondered:

*“I have a unique mind, somewhat gifted in music, mathematics, and wordplay. I have a lot of empathy, with animals as well as humans. Maybe these abilities are a result of space freed up in my brain where most people have a database of faces?”* (AF019)

However, despite these perceived silver linings to the condition, 45% of participants (13/29) could think of no benefits:

*“None at all. It’s rubbish.”* (AF006)

### 4.3 Theme 3: Widespread negative psychosocial impact

This theme captures the negative social and emotional impacts of living with DP. Although we did not directly ask about the psychosocial impact, responses to open-ended questions consistently highlighted that DP presented both cognitive challenges requiring considerable effort from participants to learn and recognise faces (see Theme 2), and also negatively impacted all participants emotionally and psychologically, at least to some extent. This impact was felt across multiple domains of participants’ lives including social relationships, restricted social development, family life, school, and career:

*“This condition impacts every aspect of my life - my relationships, friendships, work, activities and social life. It deserves more attention as it can have a seriously detrimental impact on the physical and mental health of those with the condition.”* (AF125)

#### 4.3.1 Sub theme 3.1 This is emotionally hard.

Participants commonly reported that knowing they were going to have to attempt to recognise others, e.g., at a work meeting or socially made them feel “*nervous*”, “*awkward*”, “*uncomfortable*”, or like “*a rabbit in the headlights*” and often resulted in feelings of “*anxiety*”.

*“Any occasion, social, or work, where one is expected to mingle and meet new people is hard; I can’t tell whether someone is a new person or not, so I can’t ever introduce myself to anyone (it might turn out we’ve worked together for 10 years).”* (AF003)

For many, these feelings were much stronger than the “*mild embarrassment*” reported by one participant, and negatively affected many aspects of their social life. For example:

“*I avoid groups of more than one or two people and feel very nervous in a group…. It is a shame as I love being with people… I rarely take classes or join groups with a hobby, because groups make me panic, it’s a big amorphous blob with lots of heads*.” (AF125)

#### 4.3.2 Sub theme 3.2 Isolation and social withdrawal.

“*Social occasions are terrifying and exhausting. I am warm and friendly, just exhausted from trying so hard and very, very worried*.” (AF125)

As a result of finding social situations so emotionally challenging, many participants reported often withdrawing, when possible, from social situations leading to a social life that was rather “stifled” as a result of their condition. Others said they deliberately restricted their social circle to a small number of close friends either because it took “*monumental effort to learn a new friend*” or because doing so limited the number of faces they were “*expected to know*” thus reducing the likelihood of “*embarrassing*” and “*frustrating*” failures of face recognition.

*“To be honest, I usually avoid eye contact with a lot of people and definitely avoid starting conversation because that will make for an awkward meeting and me blushing and becoming embarrassed…I don’t have very good strategies, mainly avoidance of interaction.”* (DA005)

*“I avoid crowds and parties.”* (AF019)

*“I keep a very small in-person network, which works well as a strategy for facial recognition. Though sometimes I do feel isolated.”* (DA012)

This sense of isolation was reported by many, though certainly not all, participants and several remarked that it had become more apparent in mid to late adulthood as they reflected on their life:

*“It is isolating and exhausting living in a world of strangers”* (AF125)

*“When I’m thinking back now, I realise how isolating this has been. I would meet people, talk to them and maybe have a real connection with them, but then meeting them again, I’d talk to them as if I hardly knew them until I, maybe, managed to identify them.”* (DA018)

Some participants believed that avoidance of social gatherings while growing up had also contributed to relatively underdeveloped social skills:

*“I am unsure if I was always this bad, looking back I now believe this has been a big factor in my social skills which are rather weak at best.”* (DA028)

*“Because I’ve always avoided social situations, I haven’t had enough practice at how to behave, so I’m socially very awkward and often say the wrong thing, or act inappropriately because I wasn’t properly socialised as a teenager.”* (AF006)

Although the vast majority of participants perceived that their conditions negatively affected their social and work relationships, such feelings were not unanimously expressed, one participant felt that that DP “*hasn’t affected relationships with closer friends and immediate colleagues.*” (DA009)

#### 4.3.3 Sub theme 3.3 Negative impacts on family relationships.

For some participants, the impact of DP extended beyond relationships with friends and colleagues to their family relationships:

*“My husband who I have been married to for 20 years, surprised me at the airport and I didn’t recognise him…”* (DA002)

*“I was accused by an ex-boyfriend of being racist because I didn’t recognise him when I bumped into him unexpectedly in the street, and he said ‘I suppose we all look the same to you’. This was pre-diagnosis, I didn’t know what prosopagnosia was, so I didn’t have a reply, and just felt awful because I thought he must be right.”* (AF006)

One participant wrote movingly about how DP meant they were unable to develop a close bond with their extended family, echoing the beliefs reported above that DP had limited some participants’ social relationships:

*“My dad was from a large Irish family…and I spent summer holidays in Ireland as a kid. I could never tell my many relatives apart, which I think affected my development of relationships with them. Now my dad has passed away, I desperately want to feel connected with the family, I miss being a part of it, but don’t feel I was ever able to get to know people properly so can’t reconnect now in my 40s.”* (DA001)

Furthermore, multiple qualitative responses confirmed the quantitative results that recognition of immediate and wider family can also be affected (see section 3.2 Can DPs recognise close friends and family?). For example:

*“Whilst I see faces and their features, they do not stay in my mind and I cannot easily see their face in my mind until it has permanently imprinted on my brain. I can just about recall the faces of my children but that is all. Everyone else has a blank face in my mind.”* (AF125)

*“Find my own children hard to tell apart in baby photos.”* (AF001)

*“I have consistently struggled to recognise several people [on my own doorstep] including my sister who have turned up unexpectedly.”* (DA013)

Another participant who, despite responding that they *could* recognise their immediate family, later described an instance when they had not recognised their son when he came unexpectedly to meet them in a local shop. It is possible therefore that the proportion of DPs who can reliably recognise immediate family is lower than reported, either because of faulty recall or because being unable to recognise one’s own family is perceived to be socially unacceptable as the quote below illustrates:

*“I would never dare to tell my husband, who now knows about my problem, or my sons that there are times when I have not recognised them…”* (DA018)

The extent to which participants reported receiving emotional and practical support from family members was again very mixed. Many reported that their partners and family were “understanding”, often quietly reminded them who people were, and made efforts to research the condition:

*“My family are supportive and never make me feel uncomfortable about me not being able to recognise faces.”* (DA005)

However, around 20% (6/29) of participants also reported being “teased” about their difficulties recognising familiar faces and family members were not universally supportive:

“*My close family sometimes tease me about it but are generally supportive.”* (DA022)

*“Close family not really interested and tend to think I’m making a fuss about nothing, apart from my dad (now passed away) who was delighted to find we had it in common. Partner initially thought I was being overly melodramatic, but I think is gradually coming to understand how distressing it is to realise after chatting to someone that it wasn’t who you thought it was, and you’ve been talking about things they have no clue about.”* (AF006)

### 4.4 Theme 4: Worrying what others think

Themes two and three capture just how difficult, effortful, and emotionally draining the vast majority of participants found the business of navigating everyday life. Analysis of responses revealed that these challenges often appeared to stem from pervasive worries, usually founded in prior experience, about being negatively judged by others. Theme four captures how important the positive regard of others is to DPs. It contains examples of the widespread and prominent concerns that almost all DPs expressed about potential and actual negative evaluation from others, a deep-seated worry that others would misinterpret their difficulty recognizing others for rudeness, being stuck up, cold, or uncaring. Or, perhaps worse, others might make generalisations from their face memory problems and infer that DPs were “incapable”, “stupid” or even suffering from dementia. This theme captures both participants’ *anticipation of negative reaction*, i.e., guessing how others might interpret and judge their difficulties and examples of when *participants directly experienced negative reactions,* either because they had failed to recognise familiar people or in relation to disclosing their prosopagnosia to others. Participants’ experience of receiving negative evaluation may well account for the anticipation of further negative judgement that was so prevalent among the responses.

#### 4.4.1 Sub theme 4.1 Anticipating and receiving negative evaluation causes concern.

The majority of participants perceived a strong social expectation that they should be able to recognise and therefore acknowledge people they had previously met and were aware that their condition meant they often violated these expectations. For many participants, it was feeling *“pressured to recognise people I’m supposed to know” (*AF125), rather than the cognitive difficulties associated with DP, which presented their greatest difficulties:

*“I would say the most challenging [aspect of having DP] for me is when people seem put out by the fact I don’t recognise them and they think I should.”* (AF009)

*“I hate the beginning of parties when I’m introduced to someone…I try to memorise their name and what they are wearing so I don’t offend them later on by not recognising them”* (DA001)

This sub theme was one of the most widespread, more than two thirds of participants expressed worries that their face recognition difficulty would be misinterpreted leading to negative evaluation from others. For example:

*“If I think I recognize someone I always face a dilemma - should I smile and say hello (and risk being taken for a weirdo) or ignore them (and risk being thought stand-offish)?”* (AF099)

*“…people think I am snooty as I don’t say hello to them because I don’t recognise them…”* (DA033)

*“I am naturally a sociable person, but I worry that I come across as stand-offish, e.g., when I don’t recognise people in the park or the playground.”* (DA001)

A few participants directly reported that this concern that they would be misjudged as uncaring, stupid, or rude led to anxiety or reduced self-confidence in social contexts linking back to subtheme 3.1:

*“I have anxiety in social situations because I constantly think there are people there thinking I’m rude because I don’t approach anyone, ever.”* (DA002)

*“I worry that people will think I am rude or that I don’t care about them. I feel anxious when I know I will meet a lot of new people, e.g., new job/new social activity”* (DA022)

*“It massively affects my confidence when I’m in new situations as I know I will either come across as a bit dim or forgetful or cold and rude.”* (AF125)

Additionally, one participant described how the worry itself exacerbated her face recognition difficulties and this insight may explain why some participants seem to perform better on computerised laboratory tests where the possibility of immediate, direct, negative social evaluation is absent than they do in daily life:

“In a group setting, it’s often useful to walk *away* from someone, even look at them from behind and from some distance away, because I often find that worrying about their face distracts me from using the strategies that do work for me - if I watch how someone walks, from behind, that will often settle it!” (AF003)

Although most participants reported worrying about what others might think in the context of in person social interactions, this could at times also extend to online social communication:

*“I worry that I over-compensate in non face-to-face situations, e.g., chatting on the WhatsApp group - paranoid that I talk too much, or come across as too dominant, as I’m really just overcompensating for those face-to-face interactions.”* (DA001)

Interestingly one participant reflected that they believed they experienced *less* negative evaluation when they disclosed their face recognition difficulties to others:

*“Mostly, I feel that it’s a good idea to have told people, because then if later I fail to recognise them in a way which someone might find insulting, even if I have to remind them about my prosopagnosia, it goes better if they have a vague memory that I told them this before. (If you wait until someone is already feeling insulted and then mention prosopagnosia, I think it can just come over as an excuse.)”* (AF003)

#### 4.4.2 Sub theme 4.2 Mixed social reactions to disclosure.

Initially, there appeared to be a tension between participants’ reticence to openly disclose their condition (see Fig 4), something that might be thought to reduce the widespread fear among participants that they would be misjudged (as illustrated by the previous quote). However, a possible explanation for this reluctance on the part of many participants to tell others about their face recognition difficulties may be rooted in their past experiences of disclosure. Participants who had disclosed their suspected or confirmed DP status were asked about the kind of reactions they received, and they reported a wide range of responses to disclosures of DP, whether officially diagnosed or not.

Overall, most participants had experienced a sympathetic and supportive response from at least *some* of those who they told about their difficulties; however, this was by no means a universal experience and many participants perceived that their disclosure was met with a degree of suspicion and disbelief. Around 34% (*n* = 10) experienced supportive responses, and 31% (*n* = 9) felt believed and affirmed:

*“Very surprised and interested in the condition but usually accepting and supportive.”* (DA014)

*“My family are supportive and never make me feel uncomfortable about…not being able to recognise faces”* (DA005)

However, positive reactions to disclosure of DP were by no means a universal experience. Negative reactions were also widely reported (*n* = 8), including from close family and many participants perceived that their disclosure was met with a degree of suspicion. Participants reported scepticism or even “disbelief” both that such a condition exists, and/or that the participant has it. Further, some participants (*n* = 2) reported *“very little response or acknowledgment” or a “lack of response”* to their disclosure, suggesting that they had hoped for a more positive reaction, or at least some acknowledgement of their difficulty.

*“Not sure that friends or work believe that it is a proper condition.”* (AF010)

*“My mother didn’t believe me”* (DA020)

*“My manager at work was horrible and said impatiently “honestly! We ALL don’t recognise people from time to time, it’s nothing unusual”…Partner initially thought I was being overly melodramatic, but I think is gradually coming to understand how distressing it is to realise after chatting to someone that it wasn’t who you thought it was.”* (AF006)

Others described mixed responses to their disclosure with some people being ‘*sceptical’* or treating the difficulties as a “*running joke.*”

*“Most people I have told have been interested and curious about my experience, with a few exceptions, ranging from dubious to dismissive…My youngest brother was very dismissive and convinced this is just in my head.”* (AF009)

Telling others about their difficulty was clearly not always an easy decision, one participant talked about “*coming out*” to her friend, suggesting perhaps some sense of shame and also an awareness that she had been deliberately concealing her condition for some time. Another reported that she usually only told people she’d known for some time and who she felt would believe her. Even when others had tried to understand, several participants reported feeling misunderstood due to “*misplaced sympathy*”, by which they meant that others attempted to show understanding but, in so doing, demonstrated that they had failed to fully comprehend the difficulties the participant was describing:

*“Often, people don’t understand at first (and have not heard of it) and think I am saying that I forget names.”* (DA022)

### 4.5 Theme 5: Appropriate support helps, but is often unavailable

This theme captures participants’ recommendations for things that others can do (rather than strategies that individuals with DP can try themselves) that make navigating life as a DP easier and experiences of requesting or receiving such support. This includes informal support. Although some DPs were able to suggest sources of information and advice they had personally found useful (S6 Table), it was notable how many participants were simply unable to recommend any sources, suggesting a lack of information and support for the condition. This theme also captures the other side of the coin from theme 4, instances of others being supportive and understanding of DPs’ difficulties and how valued this is.

#### 4.5.1 Sub theme 5.1 Support helps to navigate life with DP.

Most participants described instances when others had been supportive and understanding of their difficulties and emphasise how valued such support was:

*“There’s this one colleague who understands and says “Hello, it’s [his name]” every single time we meet - and every single time I am grateful. This is too awkward for most people.”* (AF003)

*“My colleagues are good are introducing other people or telling me before a person comes up who it is…”* (DA013)

*“My best friend…now goes out of her way to let me know if she has done something different to her hair when I visit.”* (AF017)

*“There is one meeting I regularly go to, in which after I mentioned it, the administrator in charge asked if it would be useful if she emailed me a chart of who is sitting where, each time. It is fantastic - by ten minutes into the meeting, I have an ascii art chart of who everyone is.”* (AF003)

#### 4.5.2 Sub theme 5.2 Reluctance to request or provide adaptations and accommodations.

Although many of the suggestions such as name badges or introductions would be simple and low cost to implement, participants noted a frequent unwillingness (with notable, greatly appreciated exceptions) on the part of colleagues, organisations, and others to implement helpful adjustments:

*I have been known to ask for, or ask the facilitator of a meeting to do a round of introductions, even when in principle everyone knows one another, just to help me. (But there is always the odd colleague who just says “oh everyone knows ME”!)* (AF003)

*I asked for name badges at meetings but no-one else was keen… it seemed a bit unnecessary for everyone else as they all knew who each other was (I had no clue who most of them were!)* (AF125)

*“It was treated as a joke. I was too embarrassed to pursue it.”* (DA017)

Analysis also indicated a reluctance on the part of some DPs themselves to request accommodations, for example at work:

*“I have not had the confidence to ask for any specific adaptions in my corporate job, other than sometimes asking team members to introduce themselves to clients first, so that I can hear the client’s name….I requested that [not-for-profit organisation] provided name badges in large capitals for the annual…conference, which they did. The request was received well. As an organisation founded on principles of equality, diversity, and human rights, I felt comfortable making this request. I probably would have felt less comfortable if it were a commercial organization.” (*DA001)

*“It doesn’t seem widespread enough to change how people operate. It’s not a visible disability so people can’t adapt to accommodate you.”* (AF075)

Some participants negatively contrasted this absence of reasonable adjustments with accommodations they observe being routinely put implemented for neurodivergent colleagues and people with visual impairments.

*“It would help if 1) People wore name badges, 2) People addressed each other by name, 3) People told me their names every time they spoke to me. 3 would be most effective, but unlikely to happen in normal life. I have experienced it in group meetings where blind people are present, and it helped me a lot.”* (AF019)

#### 4.5.3 Sub theme 5.3 Increased awareness to reduce anxiety.

Top of the list of things that participants felt would make life with DP easier were greater public and professional awareness and understanding and “recognition of the condition” particularly in schools and health care settings:

*“Training for teachers, health professionals etc. to mention prosopagnosia would be useful - especially with some idea of prevalence, because I think it’s a lot more common than most people (with or without it!) realise, because most people tend to keep quiet about it or even not know there’s a name for their difficulties.”* (AF003)

*“Training for health professionals, wider public awareness…If other people learned about it, so that we could just say that I might not recognise you if we meet again, as I have Prosopagnosia - and they would understand. If they knew that it’s not because I don’t care about them or find them important and that I don’t have dementia.”* (DA018)

*“Get it recognised by HR professionals as on a par with neurodiversity and provide advice on how they can get managers to understand the condition and what people need at work - and that they are not stupid or rude when not recognising anyone.”* (DA006)

#### 4.5.4 Sub theme 5.4 Seek advice from experts.

Although some participants were able to suggest sources of information and advice they had personally found useful (see S5 for a complete list), it was notable that the majority (19/29 or 65.5%) felt unable to recommend any sources, suggesting a dearth of information and support for the condition.

*“The majority of current resources I’ve found are inadequate. Many do not chime with my personal experience.”* (DA012)

Among the participants who did recommend sources they had personally found useful, the importance of seeking advice from others with particular expertise and knowledge around face blindness was highlighted. This could be informal peer support from individuals with lived experience of DP, unspecified Facebook self-help groups as well as from DP researchers and voluntary organisations. Several participants mentioned the support organisation Faceblind UK, and the research website Faceblind.org as useful sources of information and advice for individuals with DP and their families, however not all participants were aware of such resources.

*“The Facebook groups. Reading other peoples’ situations that I recognise so well. It has been quite liberating. Until a few years ago, I didn’t know there was such a thing as prosopagnosia! I always thought that it was “my own fault” somehow. I would definitely recommend these kind of groups.”* (DA019)

*“I possibly would recommend an official diagnosis for someone young, as this may help with coping strategies.”* (AF009)

## 5 Research priorities

Finally, participants were asked to identify their priorities for future research. They first provided spontaneous suggestions and were then asked to prioritise their top three areas for future research from a list that participants had raised in screening interviews and that researchers drew from previous grey and published literature – or to select “Other” and provide their own (Fig 7 below). Participants were also asked which of the topics they considered to be low priority.

**Fig 5:**
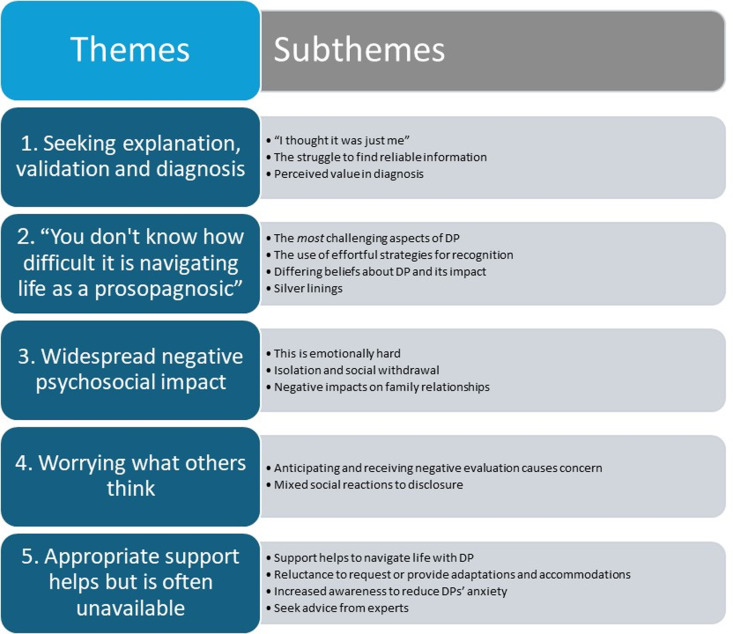
Themes and sub themes.

**Fig 6:**
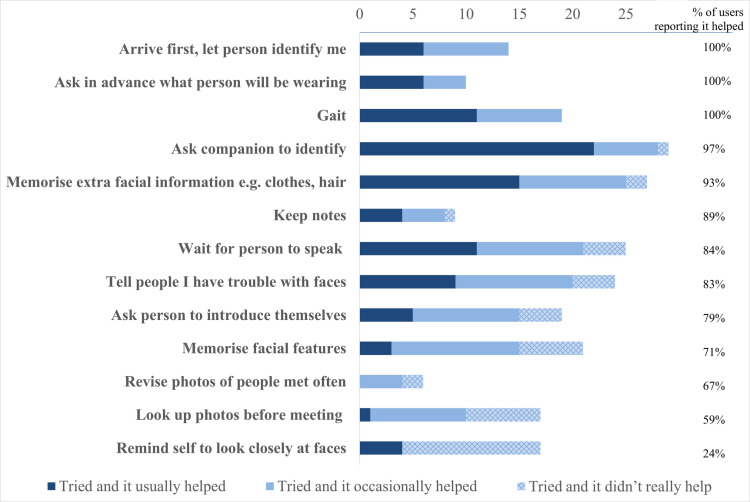
Compensatory strategies rated for usefulness by participants. *Note*: Bars show count. The value shown at the end of each bar is the proportion of those participants who had tried the strategy who reported they found it helpful, at least some of the time. For each listed strategy participants selected one of five possible responses: ‘Tried and it usually helped”; ‘Tried and it occasionally helped’; ‘Tried and it didn’t really help’; ‘Haven’t tried’, ‘Prefer not to say’.

**Fig 7:**
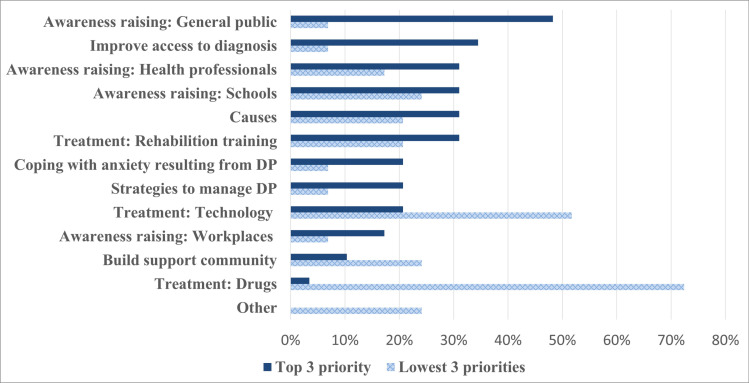
Participants’ priorities for future research. *Note*: Participants selected their top three and bottom three priorities for future DP research from a closed-choice list, or provided alternative priorities under “other”.

Participants’ *unprompted* suggestions for future research included the association and co-occurrence of DP with other conditions and factors, for example navigational ability, object recognition, personality type, sexuality, Autism, ADHD, and the ability to detect lying (see S4 Table for a full list). Most participants listed multiple avenues for future research they would like researchers to prioritise, only one participant did not feel further research was important, writing *“I don’t think it’s necessary. It’s a minor quirk, causing some social embarrassment which can be countered with more public awareness”* (DA017).

However, few of the spontaneous suggestions were later rated by any participant as being one of their top three priorities (Fig 7). Improved awareness and understanding of DP among the general public, health and education professionals, and, to a lesser extent, workplaces were highlighted as important areas for future research, followed by improved access to diagnosis. The latter perhaps reflects the difficulties that many participants had experienced having their difficulties acknowledged and accepted (see Theme 4). Additional areas participants rated as important for future research included understanding the cause(s) of DP and developing training to help improve face recognition. A couple of participants noted that they had not selected certain topics, for example developing treatment to improve face recognition, due to a belief that improvement was not possible. Others noted that they felt all of the topics were important priorities and they had struggled to choose only three.

## 6 Discussion

Twenty-nine adults with DP completed an online survey comprising closed-ended questions describing their experience of living with poor face recognition, how this manifests in their day-to-day life, the psychosocial and practical consequences of their condition, and their priorities for future research in this area. Their responses revealed that the condition affected participants in quite different ways. Some participants were slow to learn faces, needing many encounters before having any chance of recognising the person again but once a face became ‘imprinted’ in memory it was recognised reasonably well thereafter. Others were sometimes able to recognise faces after relatively few encounters, usually as a result of adopting effortful strategies, but could experience very rapid fading of face memories, after a matter of hours – or even minutes. 41% of participants reported that they were unable to reliably recognise their three closest friends out of context, and around 35% reported being unable to recognise immediate family thus highlighting that DP commonly affects the recognition of highly familiar faces with whom individuals have a close emotional relationship. Furthermore, participants who reported being able or unable to recognise their immediate family showed no significant difference in objective face memory ability.

### 6.1 Lived experiences of DP are extremely varied

The qualitative findings provided strong support for the idea that DP is a heterogeneous condition. Variability in the types of difficulties and errors that participants reported making could be due to bias or recall errors, but these factors do not appear likely to account for all heterogeneity since some participants reported directly opposing difficulties. For example, 13/29 participants said they made false alarms, i.e., mistook a stranger for someone they know, “very” or “quite” often, by contrast 3/29 said they *never* made this type of error, and false alarms are easy to detect. Most participants said they forgot familiar faces that they had *previously been able to recognise* if they hadn’t seen them for a while, however 2/29 said this *never* happened to them and 7/29 reported that this happened only infrequently suggesting that in these latter cases the face memory traces, once formed, were relatively robust. In terms of wider difficulties, many participants relied heavily on voice to recognise others, suggesting (relatively) unimpaired voice recognition consistent with previous research [41], but some found it difficult to recognise others by voice as well as by face, a finding previously reported by Liu and et al. [42]. Together, the range of types of errors, and the lack of consistent symptom presentation across the sample support the idea that DP is indeed a heterogenous condition, and that the heterogeneity widely reported in the literature [15,16,43–45] cannot solely be explained by methodological factors.

### 6.2 Face perception can be affected

Several responses indicated that, in addition to problems recognising facial identity, face *perception* was also affected in some cases of DP. For example, one participant spontaneously described a difficulty categorising facial sex, consistent with previous research [46,47] and another reported difficulty matching the face of a familiar colleague with the photo on their ID badge. Other participants described how groups of people appeared as ‘*an amorphous blob with lots of heads*” or that “*everyone merges into non people*” suggesting difficulty with face discrimination in addition to face identity recognition. Although reports of face perception difficulties were not widespread, perhaps because the questions we asked primarily interrogated face identity recognition, interestingly three separate participants spontaneously reported being extremely good at telling identical twins apart, even when their friends could not. One case of an ability to discriminate identical twins has previously been reported [20] and our finding that 3/29 participants spontaneously reported this particular strength suggests this unusual ability might be more widespread in DP than previously recognised. This suggests that the atypical techniques they employ (e.g., memorizing features or mannerisms) are effective to some extent. It was not clear from the responses whether any of the participants was able to identify either of the twins if bumping into them unexpectedly, or whether these participants were only able to discriminate the twins when seen together and in context.

### 6.3 Theoretical implications

Consistent with previous research [4,21], many participants reported finding large gatherings or busy places to be the most challenging settings; participants described attempting to locate the person they were with as being like playing the visual search task “Where’s Wally?” or recounted “losing” the face of their husband in a busy shop. Others mentioned a particular difficulty learning multiple new faces together, for example at a party. Together these findings suggest that a particular characteristic of DP is a difficulty learning or recognising faces when they are surrounded by other faces. However, the most widely used tests of face memory and face learning require participants to learn one face at a time, presented on an uncluttered background which may aid face learning. Similarly, at test, commonly used face recognition tasks typically use a single face (famous faces tests), two faces (old/new paradigms) or a three-choice paradigm, e.g., Cambridge Face Memory Test [32,48] which would not detect this specific difficulty. Two face matching paradigms, the Benton Face Recognition Task [49,50] and the 1 in 10 task [51] do require participants to identify a target face from a choice of either 6 or 10, but these tests are relatively rarely used in the DP literature [11] and such designs may force participants to rely on matching of individual features rather than the use of holistic processing which is considered a hallmark of typical upright face recognition. Interestingly a recent study identified the computerised (speeded) Benton Face Recognition Task as a *perceptual* task that was sensitive for detection of DP [52] and our findings suggest that this may because the task taps this specific perceptual difficulty that appears to be widespread among DPs. Mishra and colleagues [52] also found the Cambridge Face Perception Test [53] which requires participants to rank six faces in order of similarity to a target face at the top of a screen to be sensitive to DP; the presence of seven faces on screen might contribute to the difficulty that many [12,54], but not all [16], DPs are widely reported to experience on this task.

Although it to be expected that some individuals will believe they have particularly poor face recognition yet objective testing indicates that their abilities are not poor enough to be categorised as DP, a particular issue highlighted by several recent studies [12–14,55] is the high proportion of people who report great difficulties with face recognition yet who showed no objective face recognition difficulties, at an individual case level on laboratory tests, despite significant group level differences. Using a limited number of tasks which potentially lack ecological validity [55–57] for classification of DP may miss particular types of difficulties and our findings provide further possible explanations. For example, the use of an “emergency recognise” whereby a feature memorisation technique allows a memory to be created that can last a few hours before rapidly decaying is a strategy that could allow participants to achieve what appears to be normal face recognition scores on tests of short-term face memory such as an old new task or the CFMT.

Interestingly one participant perceived that she was better able to recognise faces as familiar at certain phases in her menstrual cycle and that during pregnancy she felt like she had a facial recognition “superpower.” Oxytocin is a neuropeptide whose levels fluctuate during menstrual cycle [58] and can increase three- to four-fold during pregnancy [59]. Face recognition ability in autism [60] and DP [61] have been associated with variations in the oxytocin receptor gene (OXTR), although other work found no relationship between autistic traits and OXTR [62]. Intra nasal inhalation of oxytocin has been reported to improve performance on face tests in people with typical face recognition [63,64] and, temporarily, in individuals with DP [65], although the latter finding has not been replicated and should thus be treated with caution.

### 6.4 Quality of life and workplace experience—A case for DP to be included in the definition of neurodivergence

With a small number of exceptions, our participants considered DP to be a lifelong condition that causes significant adverse impacts on their social and work interactions. In their personal lives participants developed bespoke ways of navigating personal and social situations, but many reported that interacting with others was a source of anxiety or even panic. Raising awareness in the general population was one of the highest research priorities listed by our participants. Campaigns raising awareness of the condition may thus be helpful in alleviating stigma or perceptions of ‘rudeness’ by people who, in fact, simply fail to recognise others. In work settings, participants struggled remembering colleagues and clients, large meetings were problematic, and some participants reported dismissive attitudes from their employers. DPs’ difficulties were particularly highlighted when work demands made it impossible to engage with compensatory strategies. The DPs’ pattern of ‘masking’ failures of face recognition strikes us as resembling masking reported by Autistic participants [66] or individuals with ADHD [67]. Masking is an exhausting and costly cognitive effort, often leading to negative mental-health consequences [68,69] and burnout [70,71].

In contrast, participants reported significant easing of pressure related to their DP by requesting adjustments such as large, capitalised name badges, or by some colleagues spontaneously helping them by repeatedly introducing themselves, or by secretaries sending them a seating plan with names. Crucially, while some participants wished that DP was recognised on par with other types of neurodivergence such as Autism and ADHD, recognised in the UK by The Equality Act 2010 [30], only one (DA001) reported feeling comfortable requesting workplace adjustments and, even then, not in their main corporate role – only in a non-for-profit organisation which espoused strong inclusivity values. It is thus clear that DP can disadvantage people in work settings (see also [72]) and that people with DP, should they wish to disclose it, should be afforded reasonable adjustments and be protected from possible discrimination. ACAS [73], the UK-based independent (but government-funded) public body providing advice on employment rights and best practice and policies includes Autism, ADHD, dyslexia, and dyspraxia in their neurodiversity [Sic] definition. We believe that DP should be included in this and similar lists of forms of neurodivergence owing to its high occurrence as a standalone condition affecting 2–4% of the population (for a review see [10]) and frequent co-occurrence with Autism [27]. While not all DPs will opt for asking for workplace adjustments, some would clearly benefit from reasonably easily implemented changes to practices.

### 6.5 Limitations and directions for future research

Although participants believed their face recognition difficulties were lifelong, we did not conduct brain scans to rule out lesions which could indicate the acquired form of prosopagnosia.

Our approach of asking open ended questions highlighted some new avenues of research that we might otherwise not have considered. For example, the findings show that individuals with DP have a particular difficulty with learning and recognising faces when multiple faces are encountered at the same time. One promising avenue for future experimental research could be to investigate whether a face recognition paradigm that presents the target face alongside distractors at study, and tests recognition among a higher number of distractor faces (including target absent trials) might be a more sensitive measure for detecting DP than current tests. Another might be to more systematically investigate DPs’ face perception abilities using a between (DP) participants approach since the individual differences in reported face perception abilities were striking. At one end of the spectrum three participants spontaneously reported being able to differentiate identical twins yet to another participant, groups appeared as a “*big amorphous blob with lots of heads.*” Additionally, research into current levels of awareness and understanding of DP among the general public and key professional audiences is warranted since participants’ top priorities for future research were awareness raising and improving access to diagnosis.

## Supporting information

S1 Table Survey questions. (DOCX)

S2 Table Correlational analysis examining relationship between real life measures and test scores. (DOCX)

S3 Table Independent Samples t-Test comparing scores of participants who do (Group = Yes) and do not (Group = No) recognise immediate family. (DOCX)

S4 Table Participants’ spontaneous suggestions for future DP research. (DOCX)

S5 Table Overview of themes, sub themes and associated codes. (DOCX)

S6 Table Recommendations from DPs for sources of advice and information. (DOCX)
