# Supplementary material for: This condition impacts every aspect of my life: A survey to understand the experience of living with developmental prosopagnosia
Source: PLoS One. 2025 Apr 30;20(4):e0322469. doi: 10.1371/journal.pone.0322469 (PMC12043184; doi:10.1371/journal.pone.0322469)
Supplement: S1 Table — (DOCX) [file pone.0322469.s001.docx]

Judith Lowes^1^*, Lesley McGregor&^¶^, Peter J.B. Hancock^1¶^, Bradley Duchaine^2^, Anna K. Bobak^1¶^

^1^ Psychology Division, Faculty of Natural Sciences, University of Stirling, Stirling, Scotland, United Kingdom

^2^ Dartmouth College, Department of Psychology and Brain Sciences, Hanover, New Hampshire, United States of America

**Supplementary materials**

**S1 Table Survey questions**

Please answer as accurately and honestly as you can. If you prefer not to answer any of the questions please tick this option or write “prefer not to say”.

Section 1: Your experience of living with face recognition difficulties.

1. Please describe how you try to recognise people you know (colleagues, family, friends, acquaintances). [open text response]
2. Please tell us what you find hardest, if anything, about having trouble recognising faces? Are certain settings more challenging, has it affected your relationships, social or work life? [open text response]
3. Please tell us about any positives, if any, about having trouble recognising faces? [open text response]

Thank you. The following questions ask you to provide some numbers, please answer as accurately as you can, even if it is an estimate.

1. Approximately how many people that you know can you reliably recognise by their face alone when meeting them in context e.g., at a large family gathering, at a regular social or sports club you attend , at college, at work? You may want to list the people on a piece of paper, count them up and enter the total here.
2. Approximately how many people can you reliably identify by their face alone if you bumped into them unexpectedly and out of context e.g. in another town? If none write 0
3. Can you always recognise your immediate family (e.g. partner/children/parents/siblings) if you meet them unexpectedly out of context? *Yes/No/Don’t know/Prefer not to say*
4. Can you always recognise your three closest friends if you meet them unexpectedly out of context? *Yes/No/Don’t know/Prefer not to say*
5. Approximately how many times do you typically need to meet a person before you might recognise them (if you never recognise anyone, please state never)?
6. Thinking about different situations where you need to recognise someone, do you find recognising a familiar face more difficult: *In a crowd/One to one/No difference/Don’t know/Prefer not to say*
7. Do you find recognising a familiar face more difficult: Indoors/Outside/No difference/Don’t know /Prefer not to say
8. Is there a setting or situation in which you find it particularly challenging to recognise faces*?* [open text response]

Next, please rate how the following statements apply to you:

1. I tend to forget familiar faces if I don’t see them regularly: Very often, quite often, occasionally, never/ Don’t know /prefer not to say
2. I can easily recall facts about people I know e.g. their pet, where they went on holiday*: Very often, quite often, occasionally, never/ Don’t know /prefer not to say*
3. I have mistaken a stranger for someone I know: Very often, quite often, occasionally, never/ Don’t know /prefer not to say

Section 2: Your experiences of seeking help and diagnosis.

Thank you. Next we are interested to hear about your experiences of seeking help or support for face recognition difficulties

1. As far as you know, have you had trouble with faces your whole life? *Yes/No*
   1. If **yes**, have you been formally diagnosed with the lifelong form of prosopagnosia (developmental prosopagnosia)? *Yes/no/Don’t’ know/prefer not to say*
      1. If yes who provided this diagnosis e.g. NHS neuropsychologist, private neuropsychologist, neurologist, university researcher, GP, Optometrist, other (please state occupation of the person who diagnosed you)
   2. If **No**: Did the difficulty recognising faces start suddenly after previously normal face recognition ability? *Yes/no/Don’t’ know/prefer not to say*
      1. if yes, please describe what happened immediately before first noticed you suddenly had trouble recognising faces e.g. head injury, illness, stroke OPEN TEXT
2. Has anyone ever suggested that you get tested for prosopagnosia/face blindness *Yes/No Don’t know/prefer not to say*

- 1. If yes, who ?tick as many as apply: Family member, friend, GP, Neurologist, neuropsychologist psychologist, eye health professional, teacher, educational psychologist, other (state)

1. And have you ever sought a diagnosis or requested to be tested for prosopagnosia? *Yes/No Don’t’ know/prefer not to say*
   1. If yes, who did you approach: GP/Teacher/Educational psychologist/Neuropsychologist/Eye health professional e.g. optometrist, optician, orthoptist/university researcher/Other (please state)

b) Please describe what happened/the response (e.g. did the professional you approached know about prosopagnosia, were you offered support and advice, or tested for prosopagnosia?) [open text response]

1. Have you every sought information/advice on prosopagnosia? *Yes/No*
   1. If yes… where from: GP, School, education psychologist, NHS Website, NHS (please state which part/service), Google, University (please name), charity (please name), Online support group (please name), social media, OTHER (please state)
2. Have you told any of the following that you have or think you have prosopagnosia? Tick all that apply:

- Spouse/partner
- Close family member
- Extended family
- Teacher/lecturer
- Colleague
- Manager
- GP or Doctor
- Close friend
- Acquaintance
- Counsellor/psychologist
- Other (please state)
- I have not told anyone

If yes, what was their response? Please state who you are referring to

1. What sources of information and advice about prosopagnosia you have you personally found particularly useful and would recommend to others, if any? [open text response]
2. What would you recommend as a first source of advice and information to a **parent** of a child or **family member** of someone with prosopagnosia, if any? [open text response]
3. What do you think would most make life easier for people with prosopagnosia e.g. information for schools, practical changes, new rules? [open text response]

Section 3 The next questions ask about strategies you use for managing face recognition difficulties

1. Please describe any strategies you use to help you recognise people in everyday life. Please state None if you don’t use any [open text response]
2. Some people have reported that they use some of the following strategies to help them recognise faces. Please tell us about your experience of using the following strategies: I *tried and it helped, I tried and it didn’t really help, I have not tried, prefer not to say*

- I try to memorise details about individual faces like freckles or scars
- I look up photos of people before I meet them e.g. staff directory, social media
- I ask a companion to identify the person for me
- I arrive first so that person I am meeting will find me rather than the other way around
- I ask people to introduce themselves
- I ask people to tell me what they will be wearing when we meet so I can find them by their clothing
- I take note of how people walk
- I try to wait until someone speaks so I can recognise them by voice
- I keep notes with descriptors of people that will help me recognise them again
- I keep photos of people I meet often and look over these on a regular basis
- I tell people I have trouble with faces
- I try to remember details like clothing, hairstyle, accessories, glasses
- When I meet someone, I remind myself to look closely at their face, even it if doesn’t feel comfortable

1. Thinking about your place of work or study, please tell us about any adaptations you use(d) in workplace/school/college to help you recognise people (e.g., ask your colleagues to always introduce themselves, name badges). State NA if you don’t use any [open text response]
2. Have you ever formally requested your place of work or study to provide adaptions for your face recognition difficulties E.g. using name badges? *Yes/no/Don’t know/Prefer not to say*

If yes,

1. Have you ever formally requested your place of work or study to provide adaptions for your face recognition difficulties E.g. using name badges? What response did you receive? E.g. Did the organisation agree? Were adaptations put in place? How was your request received? [open text response]
2. To what extent did you find the adaptations helped you if at all? [open text response]

Section 4: Research priorities

1. What is important to you in terms of what researchers dedicate their time to examining prosopagnosia? What would you like to see more research on*?* [open text response]
2. How would you rate the following priorities for future prosopagnosia research (order them 1-10 where 1 is top priority and 10 is lowest priority)

- Developing training to help improve face recognition
- Developing drug treatments to help improve face recognition
- Building a support community of people and families with DP
- Awareness raising for the general public
- Awareness raising for health professionals
- Awareness raising for workplaces
- Awareness raising for schools
- Improving access to diagnosis for prosopagnosia
- Strategies to help people manage/work around their face recognition difficulties
- Coping with anxiety resulting from prosopagnosia
- Researching the causes of developmental prosopagnosia
- Other

1. Finally, is there anything else you would like to share with us? [open text response]

Demographics: Please tell us about yourself

What year were you born?

Gender (open text),

Where do you live? *England, Scotland, Wales, Northern Ireland, Republic of Ireland, Channel Islands, Isle of Man , Other* (please state)

Employment status (*working, unemployed but available to work, not working due to illness/ disability or family reasons, student, retired, prefer not to say*)

As far as you know, does anyone else in your family have trouble recognising faces ? (*yes/no/don’t know/prefer not to say)*
