# Supplementary material for: This condition impacts every aspect of my life: A survey to understand the experience of living with developmental prosopagnosia
Source: PLoS One. 2025 Apr 30;20(4):e0322469. doi: 10.1371/journal.pone.0322469 (PMC12043184; doi:10.1371/journal.pone.0322469)
Supplement: S2 Table — (DOCX) [file pone.0322469.s002.docx]

Judith Lowes^1^*, Lesley McGregor&^¶^, Peter J.B. Hancock^1¶^, Bradley Duchaine^2^, Anna K. Bobak^1¶^

^1^ Psychology Division, Faculty of Natural Sciences, University of Stirling, Stirling, Scotland, United Kingdom

^2^ Dartmouth College, Department of Psychology and Brain Sciences, Hanover, New Hampshire, United States of America

**S2 Table Correlational analysis examining relationship between real life measures and test scores**


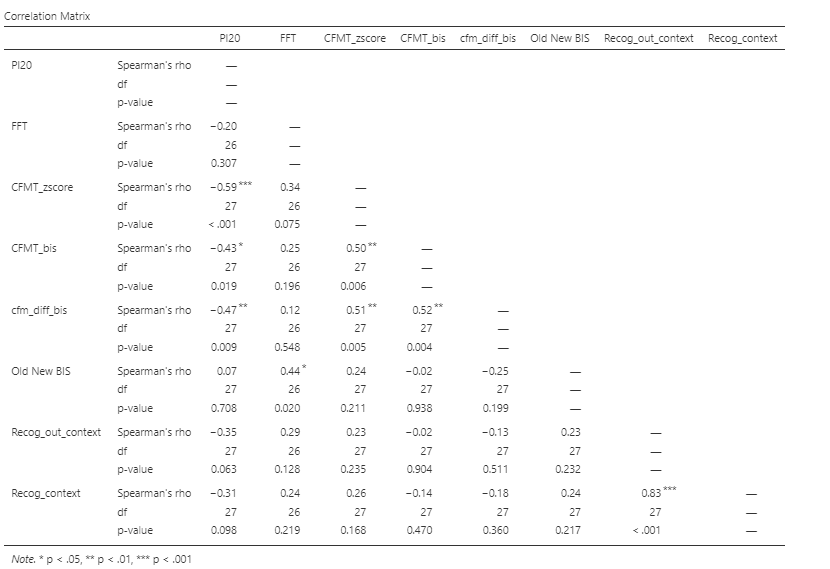


*Note*: PI20 = Prosopagnosia Index 20, CFMT = Cambridge Face Memory Test, z score _ standardised proportion correct, BIS= Balanced Integration Score, cfm_diff_bis = difference between face memory BIS and Bicycle memory BIS, FFT = Famous Faces Test, Old New = Old new Faces test, Recog_out _context = number of faces participants calculated they could recognise if meeting out of context, Recog_context = number of faces participants calculated they could recognise if meeting in context.
