# Supplementary material for: This condition impacts every aspect of my life: A survey to understand the experience of living with developmental prosopagnosia
Source: PLoS One. 2025 Apr 30;20(4):e0322469. doi: 10.1371/journal.pone.0322469 (PMC12043184; doi:10.1371/journal.pone.0322469)

Judith Lowes^1^*, Lesley McGregor&^¶^, Peter J.B. Hancock^1¶^, Bradley Duchaine^2^, Anna K. Bobak^1¶^

^1^ Psychology Division, Faculty of Natural Sciences, University of Stirling, Stirling, Scotland, United Kingdom

^2^ Dartmouth College, Department of Psychology and Brain Sciences, Hanover, New Hampshire, United States of America

**S3 Table Independent Samples t-Test comparing scores of participants who do (Group = Yes) and do not (Group = No) recognise immediate family
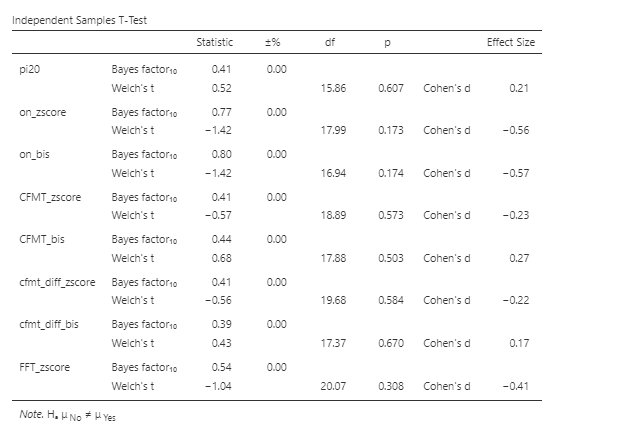
**

*Note*: PI20 = Prosopagnosia Index 20 CFMT = Cambridge Face Memory Test, z score = standardised proportion correct, BIS= Balanced Integration Score, cfmt_diff_zscore = standardised difference between face memory and bicycle memory BIS, FFT = Famous Faces Test, on = Old new faces test


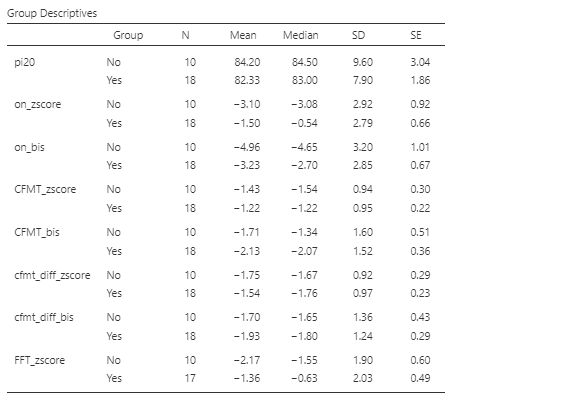

Supplement: S3 Table — (DOCX) [file pone.0322469.s003.docx]
