# Supplementary material for: This condition impacts every aspect of my life: A survey to understand the experience of living with developmental prosopagnosia
Source: PLoS One. 2025 Apr 30;20(4):e0322469. doi: 10.1371/journal.pone.0322469 (PMC12043184; doi:10.1371/journal.pone.0322469)
Supplement: S4 Table — (DOCX) [file pone.0322469.s004.docx]

Judith Lowes^1^*, Lesley McGregor&^¶^, Peter J.B. Hancock^1¶^, Bradley Duchaine^2^, Anna K. Bobak^1¶^

^1^ Psychology Division, Faculty of Natural Sciences, University of Stirling, Stirling, Scotland, United Kingdom

^2^ Dartmouth College, Department of Psychology and Brain Sciences, Hanover, New Hampshire, United States of America

**S4 Table Participants’ spontaneous suggestions for future DP research**

| **Theme** | **Suggestions** |
| --- | --- |
| **Rehabilitation of DP** | - Effective techniques for recognising people - Strategies for managing - Help for the severe cases that most likely require rehabilitation - Top tips - Can it be improved? - How to help children understand and adapt to DP - Is there a cure? - More therapeutic and/or technical strategies - What may be successful non-clinical interventions and training or therapy. - Whether you can train yourself to improve your facial recognition (similarly to how people with dyslexia can improve their reading). - Adaptations which might help - Strategies that help those with it |
| **Understanding** | - How and when does it develop? - Are there different subtypes - Gain a better understanding of the condition - Is the part of the brain that's doing face recognition badly, doing something else ? - Why this happens and in particular if there are circumstances where it is more likely to occur. - An explanation for why I struggle would be interesting - Can DP happen randomly with no trauma involved? - A better understanding of what causes it and why would be very interesting. - Why it happens - Whether there are any benefits to having prosopagnosia (eg better voice recognition). - What it is, how it develops, how it differs between people, genetic basis, - Is there any hormonal link. e.g. I find that I'm slightly better at recognising people during certain times of the month. Also whilst I was pregnant. |
| **Co-occurrence with other conditions and identities** | - Association with other conditions e.g. Autism, navigation, object recognition, synaesthesia, anxiety, dyslexia, poor direction finding, mixed handedness, ADHD, aphantasia were specifically mentioned - Links with stress and general mental load, e.g. I've noticed I find it harder to recognise faces during times of greater workload, or if I've been to an event this week where I've met lots of people. |
| **Awareness of DP** | - What are the current levels of awareness of DP in general population - Other peoples' understanding of face blindness / prosopagnosia (i.e. people who don't have it). What do they make of it? What effect would raising public awareness have on other peoples' understanding. Including in the work place. - Perhaps raising awareness would be useful - Wider discussion in the media. |
| **Early intervention** | - Mass testing of children - How and when does it develop? Are there interventions for children that would make it less likely? |
| **Other** | - I think we need as much data as possible before we can decide what the most beneficial research direction will be. So more research on the lived experience is probably a good thing in the first instance. - Sexuality, (are faceblind people more likely to be asexual?) - Are faceblind people more interested in reading and is this associated with increased empathy, focus and concentrate, better sleep and brain ageing, - Whether the condition is associated with different personality types and whether different personality types deal with the condition differently |
