# Supplementary material for: This condition impacts every aspect of my life: A survey to understand the experience of living with developmental prosopagnosia
Source: PLoS One. 2025 Apr 30;20(4):e0322469. doi: 10.1371/journal.pone.0322469 (PMC12043184; doi:10.1371/journal.pone.0322469)
Supplement: S5 Table — (DOCX) [file pone.0322469.s005.docx]

Judith Lowes^1^*, Lesley McGregor&^¶^, Peter J.B. Hancock^1¶^, Bradley Duchaine^2^, Anna K. Bobak^1¶^

^1^ Psychology Division, Faculty of Natural Sciences, University of Stirling, Stirling, Scotland, United Kingdom

^2^ Dartmouth College, Department of Psychology and Brain Sciences, Hanover, New Hampshire, United States of America

**S5 Table Overview of themes, sub themes and associated codes**

| **Theme and description** | **Sub themes** | **Associated codes** |
| --- | --- | --- |
| **1. Seeking explanation, validation and diagnosis**  This theme describes the process by which participants came to realise their face recognition difficulties were atypical, and the existence of DP as a condition. It captures the sources of information participants sought out in order to understand or explain their difficulties, and their experiences of seeking a diagnosis (or classification) whether formal, informal or self-diagnosis and how this made them feel . | 1.1 “I thought it was just me“ | Realising has DP |
|  | 1.2 The struggle to find reliable information | Internet  Media  Medical sources  Other DPs/social media  Support organisations  University researchers  Other sources |
|  | 1.3 Perceived value in diagnosis | Who was approached for diagnosis  Misdiagnosis |
| 2. ***“You don't know how difficult it is navigating life as a prosopagnosic”.***  This theme captures depictions of what it’s like to live with poor face recognition, including whether this has changed over time – for example whether certain life stages were more difficult and if the condition gets more, or less challenging, with age. It describes the settings and circumstances that participants said they find the hardest as well as any perceived advantages of DP. It captures the techniques participants use in their everyday lives to try to recognise people and the extent to which they find these helpful. It also captures beliefs participants had about the condition and these, and the condition itself , affects their self-identity. | 2.1 The *most* challenging aspects of DP | Main challenges  Challenging settings and situations  Factors that exacerbate difficulties  Factors that exacerbate difficulties: Cognitive load  Work experiences |
|  | 2.2 The use of effortful strategies for recognition | Body  Clothing  Gait  Hair  Mannerisms  Memorise features  Other strategies  Paraphernalia  Prompts from others  Situational context  Staged approach  Stringing conversation along  Use of notes  Voice  Wait for others to speak first  Effortful  Strategy failures |
|  | 2.3 Differing beliefs about DP and its impact | Age and stage  Surprise oneself  DP as a disability  Severity  Self-identity  Lack of agency  Acceptance  Coping strategies |
|  | 2.4 Silver linings | Advantages of DP  Provides perspective  Positive affect |
| **3.Widespread negative psychosocial impact**  This theme captures the varied aspects of life that are affected by DP and how the condition negatively impacts the lives of many participants; emotionally and psychologically, in relation to restricted social relationships and social development, family life, school and career. This theme also highlights that DPs often struggle to recognise those they are closest to, family and friends as well as colleagues they see very regularly and know well e.g. at work. | 3.1 This is emotionally hard*.* | It’s hard  Negative emotions  Feelings of embarrassment |
|  | 3.2 Isolation and social withdrawal | Effects on social relationships  School experiences  Social circles are kept deliberately small  Perception that social skills are underdeveloped  Social withdrawal and isolation  Affects ability to believe others |
|  | 3.3 Negative impacts on family relationships | Impact on family relationships  Family history of poor face recognition |
| **4. Worrying what others think**  This theme captures how extremely important the positive regard of others is to DPs. It contains examples of the widespread and prominent concerns that almost all DPs expressed about potential and actual negative evaluation from others, a deep-seated worry that others would misinterpret their difficulty recognizing others for rudeness, being stuck up, cold or uncaring. The sub themes capture both *anticipated* negative reaction and judgments, i.e. guessing how others might interpret and judge their difficulties and also *direct experiences of* other’s negative reactions both in relation to observed instances of failures to recognise familiar people as well as experience of disclosure. | 4.1 Anticipating and receiving negative evaluation causes concern | Worrying what others think  Fear of being considered uncaring  Fear of being thought rude  Fear of being thought standoffish or stuck up  Fear of being considered stupid or incapable  Negative reactions from others  Hostile or impatient  Teasing or joking  Others misunderstand or misinterpret |
|  | 4.2 Mixed social reactions to disclosure | Feeling believed  Not feeling believed  Supportive response  Lack of response  Surprised  Unsurprised  Tried but failed to understand |
| **5. Appropriate support helps but is often unavailable.** This theme captures participants’ recommendations for things that others can do (rather than that individual DPs themselves can do) to make navigating life as a DP easier, including informal support. This theme also captures the other side of the coin from theme 4, instances of others being supportive and understanding of DPs’ difficulties and how valued this is. | 5.1 Support helps to navigate life with DP | Supportive  Pro-actively supportive  Family and friends  Sources of support: employer  Sources of support: colleagues  Employer  Colleagues  Other sources of support |
|  | 5.2 Reluctance to request or provide adaptations and accommodations | Adaptations and accommodations  Disclosure as a strategy |
|  | 5.3 Increased awareness to reduce anxiety. | Need for awareness  Disclosure as a strategy |
|  | 5.4 Seek advice from experts | Advice for others |
