# Supplementary material for: This condition impacts every aspect of my life: A survey to understand the experience of living with developmental prosopagnosia
Source: PLoS One. 2025 Apr 30;20(4):e0322469. doi: 10.1371/journal.pone.0322469 (PMC12043184; doi:10.1371/journal.pone.0322469)
Supplement: S6 Table — (DOCX) [file pone.0322469.s006.docx]

Judith Lowes^1^*, Lesley McGregor&^¶^, Peter J.B. Hancock^1¶^, Bradley Duchaine^2^, Anna K. Bobak^1¶^

^1^ Psychology Division, Faculty of Natural Sciences, University of Stirling, Stirling, Scotland, United Kingdom

^2^ Dartmouth College, Department of Psychology and Brain Sciences, Hanover, New Hampshire, United States of America

**S6 Table**

**Recommendations from DPs for sources of advice and information^^[[1]](#footnote-2)^^**

**What sources of information and advice about prosopagnosia have you personally found particularly useful and would recommend to others with face blindness, if any?**

- Faceblind UK have a marvellous book about what it's like living with prosopagnosia, with quotes from prosopagnosics. I lent it to several people at work to help them understand.
- I realised I had face blindness after I read the young adult novel "Holding Up the Universe" by Jennifer Niven. I recommend it as it opened my eyes to face blindness and on reflection, I find it valuable to see someone like me in a book. I recommend faceblind.org and their newsletter.
- I like the little badge that I got from Faceblind UK- it’s something subtle that I wear on my lanyard and sometimes people ask.
- I would advise others to visit faceblind.org.uk, and to seek out and engage with university researchers.
- Can't think of anything specific. I think by the time you know you have prosopagnosia you tend to be an expert! The Facebook groups can be helpful but mostly for support, not information.
- From my research these isn’t a huge amount out there, but there’s a few self diagnosis questionnaires and I would recommend faceblind.org
- The Facebook groups. Reading other peoples' situations that I recognise so well. It has been quite liberating. Until a few years ago, I didn't know there was such a thing as Prosopagnosia! I always thought that it was "my own fault" somehow. I would definitely recommend these kind of groups.
- Just that I’m not alone
- I enjoy listening to the podcasts on face blindness because they provide real life experiences from regular people who are experiencing the same thing as me. I read academic articles and controlled news or university websites on the topic. I studied Psychology at university so I also have class notes on the disorder.
- I think mainly that some of the anxiety I feel in social situations is a result of face blindness, and that doesn’t' make me stupid or shallow. Otherwise, I think, just get on with it
- Other than the information offered to me by Judith I don't think I have any other advice or information offered to me.
- Don’t sweat about it! Just let people know you’re not rude. You have a neurological condition
- Faceblind UK has lots of interesting information, but I also found lots of information on random websites that I cannot remember details of. Joining the research project was also very helpful and interesting.
- Faceblind.org. The NHS page was also useful as an overview, but the info is v basic.
- Radio 4 programme which initially gave a name to this (about 12-15 years ago). Before that I had thought it was just me. Following this I found the faceblind.org website and signed up for their newsletter which I read. I have read the young adult book "holding up the universe" by Jennifer Niven which included a main character who had severe face-blindness. I would recommend this book.
- Facebook page for people with the condition

**What would you recommend as a first source of advice and information to a parent of a child or family member of someone with prosopagnosia, if any?**

- Google
- All I could suggest is they do their own research and offer to discuss their [child’s or family member’s] experiences with them
- The majority of current resources I've found are inadequate. Many do not chime with my personal experience.
- Face Blind UK is the only source I am aware of, never really thought about googling it
- Nancy Mindick's book Understanding Facial Recognition Difficulties in Children.
- BBC news article on face blindness and podcasts by academics based on scientific evidence.
- Google I think. I think don’t regard it a source of embarrassment, just treat it as something that makes you different, but not worse. And be honest with people if you don’t recognise them, say you are bad at recognising, which is what I say to people.
- I think I would now point them toward Judith! But other than that I suppose the internet would be my obvious place to turn.
- Look for a self help group or society. If it’s come on suddenly or a child involved, seek investigations from your GP to rule out anything serious.
- Face blind UK, and I possibly would recommend an official diagnosis for someone young, as this may help with coping strategies.
- With caveats, online
- I don't really know but I would say research a reliable specialist charity and contact them (if they exist).
- NHS website, Wikipedia
- Faceblind.org website (or if a child the book I mentioned "Holding up the universe" by Jennifer Niven).

1. Duplicate answers are combined [↑](#footnote-ref-2)
